# Interpretable neural networks: principles and applications

**DOI:** 10.3389/frai.2023.974295

**Published:** 2023-10-13

**Authors:** Zhuoyang Liu, Feng Xu

**Affiliations:** ^1^Key Lab of Information Science of Electromagnetic Waves, Fudan University, Shanghai, China; ^2^Faculty of Math and Computer Science, Weizmann Institute of Science, Rehovot, Israel

**Keywords:** model decomposition, semantic graph, interpretable neural networks, electromagnetic neural network, interpretability

## Abstract

In recent years, with the rapid development of deep learning technology, great progress has been made in computer vision, image recognition, pattern recognition, and speech signal processing. However, due to the black-box nature of deep neural networks (DNNs), one cannot explain the parameters in the deep network and why it can perfectly perform the assigned tasks. The interpretability of neural networks has now become a research hotspot in the field of deep learning. It covers a wide range of topics in speech and text signal processing, image processing, differential equation solving, and other fields. There are subtle differences in the definition of interpretability in different fields. This paper divides interpretable neural network (INN) methods into the following two directions: model decomposition neural networks, and semantic INNs. The former mainly constructs an INN by converting the analytical model of a conventional method into different layers of neural networks and combining the interpretability of the conventional model-based method with the powerful learning capability of the neural network. This type of INNs is further classified into different subtypes depending on which type of models they are derived from, i.e., mathematical models, physical models, and other models. The second type is the interpretable network with visual semantic information for user understanding. Its basic idea is to use the visualization of the whole or partial network structure to assign semantic information to the network structure, which further includes convolutional layer output visualization, decision tree extraction, semantic graph, etc. This type of method mainly uses human visual logic to explain the structure of a black-box neural network. So it is a post-network-design method that tries to assign interpretability to a black-box network structure afterward, as opposed to the pre-network-design method of model-based INNs, which designs interpretable network structure beforehand. This paper reviews recent progress in these areas as well as various application scenarios of INNs and discusses existing problems and future development directions.

## 1. Introduction

Human natural intelligence arises from the evolutionary innate brain after empirical learning. Human intelligence has invented computing technology, and now people hope to use it to implement artificial intelligence (AI). With massive big data and high-performance computing, the emergence of deep learning, that is, DNNs have led to the explosive development of AI. However, DNNs are still essentially a function-fitting technique. They are black-box methods lacking interpretability and have weak generalization ability when the network doesn't have enough high-quality training data. The ability to logically reason is one of the basic characteristics of human intelligence. Getting inspiration from the process of human logical reasoning to realize explainable AI is one of the directions of next-generation AI.

Human intelligence's logical reasoning can be classified as either deductive or inductive reasoning (Goswami, 2011). Deductive reasoning starts with a clear premise, which often is a well-known fact or truth. It can be used to construct a theoretical model through principles, so it has a rigorous expression (Clark, 1969; Johnson-Laird, 1999). Inductive reasoning is similar to data analysis, fitting, and clustering in that it draws on prior experience to predict current or future events (Sternberg and Gardner, 1983; Heit, 2000). As can be seen, existing deep learning (DL) approaches are analogous to inductive reasoning, that is, inducing principles from massive datasets. However, human inductive reasoning is interpretable since the process of human induction follows a well-defined semantic framework. Specifically, humans use eyes to sense the world and then use inductive reasoning to infer the type of a new thing and obtain semantic information from prior knowledge. Hence, by learning from the nature of human inductive reasoning, existing DL methods can accomplish semantic INN. On the contrary, traditional non-machine learning methods are similar to deductive reasoning, which refers to the process of developing theoretical models based on domain knowledge by specialists. Then the appropriate algorithms are developed to solve these problems by utilizing the theoretical models. Thus, interpretability can obviously be achieved by drawing inspiration from theoretical model decomposition.

**Figure 1:**
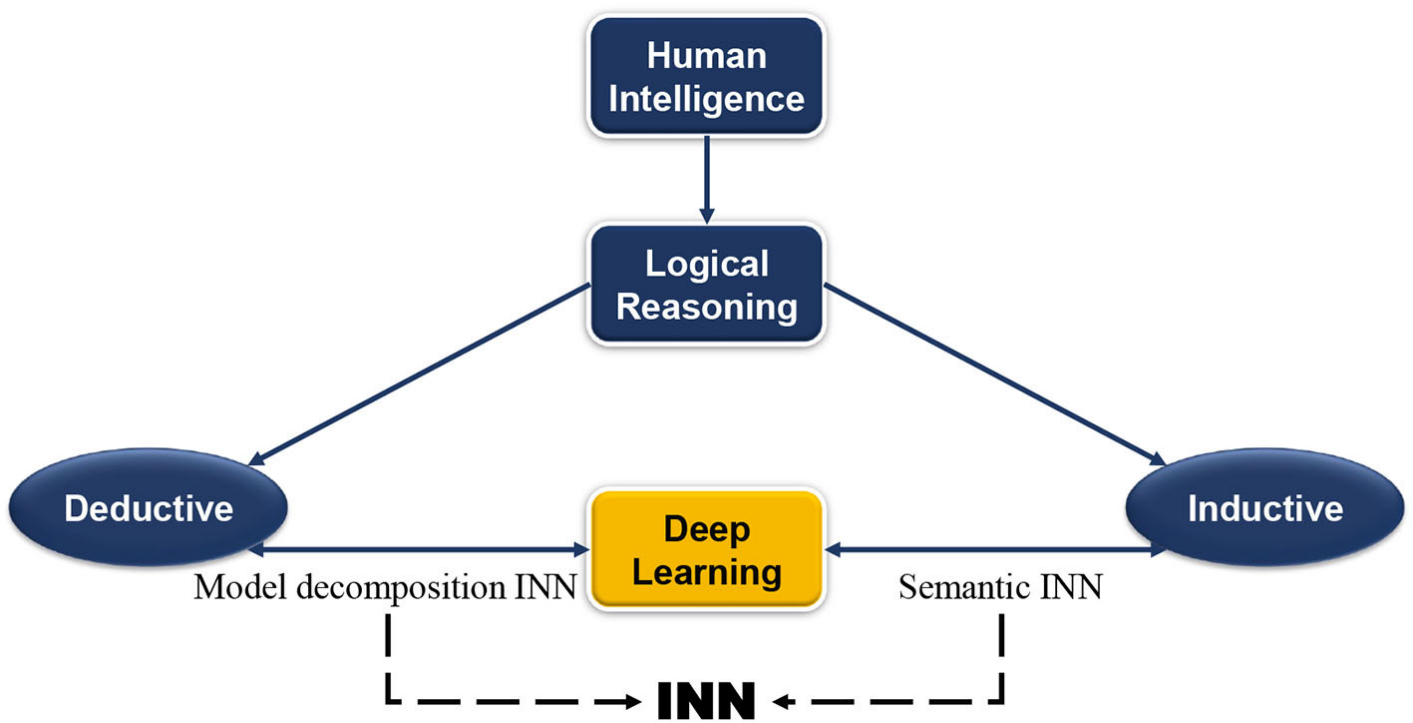
Human intelligence and artificial intelligence.

As shown in Figure 1, this paper reviews and analyzes the existing INN research according to these two ideas. Model decomposition alternative INN learns from traditional theoretical models, that is, the combination of DL models and theoretical models to realize INN with the domain knowledge embedded in the network designing. On the contrary, semantic INN is closer to human semantic interpretation, and it is the combination of DL with the process of semantic inductive reasoning, which adds clear semantic information to neural networks (NNs) afterward. These two approaches can help mitigate issues of current data-driven approaches such as weak generalizability, inexplicability, and low fidelity. The principles of defining and implementing INN are mainly discussed in this paper. This section discusses the origin of INN along with the development and limitations of DL, and finally gives its definition and development as well as the practical applications.

### 1.1. Demands and challenges of INN

**Figure 2:**
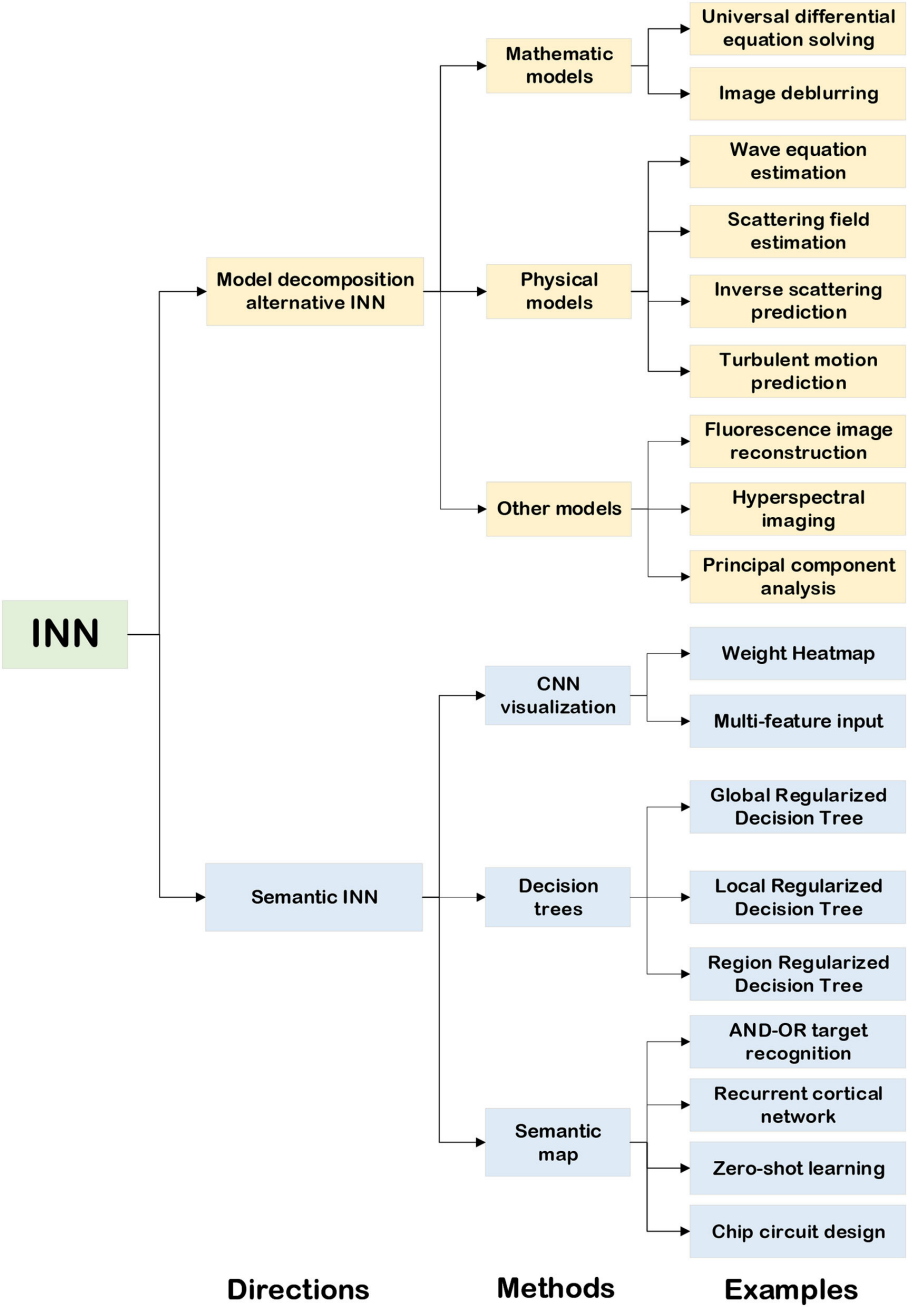
The classification of INN.

The rapid development of DNN has benefited from big data, improved algorithms, and high-efficiency computing. However, the current DL methods are completely data-driven, which means that very large-scale annotated data are required for training to get ideal results (McCulloch and Pitts, 1943; Deng et al., 2009; Dahl et al., 2011; He et al., 2015; Krizhevsky et al., 2017; Zhou et al., 2017; Montavon et al., 2018). As a black-box approach, it has serious drawbacks in terms of robustness and interpretability. In many AI-powered application scenarios such as autonomous driving, target recognition, etc., interpretability and robustness are crucial aspects of AI technology. Gregor and LeCun (2010) were the first people to put forward the theory of interpretability of neural networks. They adopted the method of combining sparse coding with traditional NNs so that DL inherited the model-based method's interpretability and the learning-based method's efficiency. According to the current research, we summarize the existing approaches of constructing an INN as two groups, which are the model decomposition alternative INN and the semantic INN, based on the way to perform inference, as shown in Figure 2.

Conceptually, the common thread of delivering applications based on the model decomposition alternative INN is related to human deductive reasoning. While implementing the model decomposition alternative INN, the investigator decomposes the conventional algorithm based on the mathematical-physical model into several calculation steps, which can be transformed into the computation process of a NN. Similarly, the common thread of explaining applications based on the semantic INN is relevant to human inductive reasoning. The implementation of the semantic INN is to construct the explanation graph with the assistance of engineers, which in turn helps them determine whether the network is working correctly. Following the above INN construction principles, Sections 1.2, 1.3 describe the application of two types of INN in detail.

### 1.2. Applications of model decomposition alternative INN

The model decomposition alternative INN is based on the facts of the real world. It decomposes a complex mathematical model or physical model into several modules that are easier to handle. Then, according to the prior knowledge, it transforms the computational process of these modules into NNs' hyper-parameters or hidden layers so that the NNs are interpretable (Zhang et al., 2018; Shlezinger et al., 2020). This kind of interpretable method is equivalent to unfolding the “black box” of the original NNs and using some artificial and controllable parameters and structures to replace the weights without mathematical and physical meaning in DNN. In order to extract these artificial and controllable parameters and structures, the problem must have a theoretical model. Applications of INNs based on mathematical models, physical models, and some other models are given in Figure 2.

For example, the mathematical modeling problem solved by convex optimization or non-convex optimization algorithms can be used to guide the designing of the objective function. This method can be used to solve common partial differential equation (PDE) (Rudy et al., 2017; Zhang et al., 2019b; Rackauckas et al., 2020) or image deblurring, super-resolution, and other tasks (Daubechies et al., 2004; Wang et al., 2015; Li et al., 2020).

The role of the physical model in model decomposition alternative INN is different from that of the mathematical model. The computing process and parameters that have physical meanings of standard algorithms solving physical models are replaced by hidden layers and weights in NNs. In the field of electromagnetic physics, Fan et al. combine the finite difference time domain (FDTD) method to construct an recurrent neural network (RNN) to model the propagation of the wave equation and estimate the medium parameters (Hughes et al., 2019). Guo et al. (2021) use the method of moment (MOM) to construct an INN, which computes the forward scattering field in a two-dimensional plane and predicts the inverse scattering parameters (Li et al., 2018; Wei and Chen, 2019; Xu et al., 2020). In the field of fluid mechanics and dynamics, Fang et al. (2021) use data-driven acceleration to deduce the speed and position of turbulent motion and used a physics-informed neural network (PINN) to discover the parameters of the higher-order nonlinear Schrodinger equation (NLSE). There are also many pieces of research using PINN to solve dynamic equations with a special INN method (Brunton et al., 2016; Sirignano and Spiliopoulos, 2018; Kochkov et al., 2021).

Other models in Figure 2 include non-mathematical physical models, such as problems in the field of biochemistry. By dealing with fluorescent images, Belthangady and Royer (2019) and Li et al. (2021) summarized applications of using DL to achieve microwave fluorescence image reconstruction. In ultrasound imaging, the intensity of clutter signals is usually relatively large and the distribution range is relatively wide, which seriously affects the accuracy of ultrasound imaging. The INN combined with principal component analysis (PCA) is proposed to achieve main beam extraction and clutter removal in Chien and Lee (2017); Lohit et al. (2019); Solomon et al. (2019). The performance of model decomposition INN is closely related to the physical limitations of the specific theoretical models.

### 1.3. Applications of semantic INN

Another direction is semantic INNs, and their interpretability mainly comes from the perspective of the human brain to realize the interpretable meaning of DNNs (Fan et al., 2021). Obviously, this is closely related to semantics, which means features or attributes described by people in language, and it reflects the process of people's understanding of the real world. Furthermore, it includes three aspects, i.e. visualization of convolution neural network (CNN), decision tree regularization, and semantic knowledge graph.

Visualization CNN is an interpretable method for a trained model. The basic idea is to display the output of each feature map of the network in the form of a weight heat map to show what role each layer played in accomplishing a given task (Wang et al., 2018a; Zhang and Zhu, 2018). In other words, it can activate different regions in the layers of NNs to distinguish the meaningful parts of the input (Guidotti et al., 2018). Its interpretability is reflected in the visualization, and the NN model itself is still a “black box.”

The methods based on decision trees are proposed to help achieve the interpretability of NNs (Frosst and Hinton, 2017; Wu et al., 2018). The decision tree is a directed graph composed of parent nodes and child nodes. Its parent nodes and child nodes have semantic information, and the directed connections of decision trees make the path between the parent node and each child node also meaningful. Combining the decision trees which are the prior knowledge with layers of NNs can enhance the interpretability and robustness of DNNs. According to the region where the regularization acts, the interpretability method of decision tree extraction is divided into three types, which are global, local, and regional regularization decision trees, respectively (Lapuschkin et al., 2019; Wu et al., 2020).

The third approach is an interpretable graph neural network (GNN) that combines semantic graphs and DNNs, and its main idea is to utilize the semantic information contained in graphs to enhance the interpretability of DNNs. Zhang et al. (2017) use the AND-OR structure to realize target recognition (Si and Zhu, 2013; Akula et al., 2019), and the knowledge graph (KG) was mapped to the convolutional layers and the pooling layers. George et al. (2017) add side connections to form a recursive cortical network (RCN) which realized the verification code images denoising. The recently emerging zero-shot learning utilizes a mixture of KG, GNNs, and DNNs, which are combined with KG to realize the function of NNs inference learning, and multi-sample detection or recognition (Lampert et al., 2009; Palatucci et al., 2009; Kipf and Welling, 2016; Wang et al., 2018b; Chen et al., 2019; Lu et al., 2019; Yue et al., 2021). In the field of integrated circuit (IC) design, Mirhoseini et al. regard the circuit diagram as a GNN and used the semantic feature extraction to complete efficient and accurate IC design (Mirhoseini et al., 2021). Chen et al. (2021) use a GNN expansion to crop an overlapped graph, extract the main parts of the graph, and realize graph denoising.

### 1.4. Comparison of model decomposition alternative INN and semantic INN

The research on interpretability is currently in the development stage. A large body of literature describes the implementation of explaining NNs and the construction of INNs. The above sections introduce two types of INN techniques and list the applications of INNs in signal processing, image classification, solving differential equations, etc. To better comprehend the basic principles of INNs, we emphasize that model decomposition alternative INN provides the mapping between the mathematical-physical model and NN's parameters or structures, while the semantic INN extracts the explanation graphs from NN by engineers using standard coding methods. The former converts mathematical-physical models that humans can understand into operators that computers can recognize, while the latter transforms the output of computers into semantics that humans understand.

Specifically, for the presented application of the model decomposition INN method, such as solving differential equations and image restoration, embedding a mathematical-physical model into the NN enhances the robustness of network training and convergence performance. However, those applications mentioned here have no semantics, so they are not reasonable for verifying network results with semantic INN analysis. Similarly, considering image classification tasks using semantic INN, extracting explanation graphs from pre-trained NNs assists engineers in evaluating the training state of NNs and improving their reliability. Still, the image classification task is hard to describe as a mathematical-physical model, thus it is not realistic to modify the network structure by embedding the traditional model. In other words, those two types of INNs are suitable for different tasks, and we need to choose the corresponding INN method according to the requirements.

To sum up, INNs are widely used in various fields. People pay great attention to the principles of high efficiency of NNs, and INN can give a reasonable explanation that ensures the reliability and security of network outputs. This paper focuses on the definition of INN and how to use INNs. In the following sections, the model decomposition alternative INNs and semantic INNs are introduced in detail. Finally, we present the application of INNs to solve practical electromagnetic problems and conclude with a summary.

## 2. Model decomposition alternative INN

**Figure 3:**
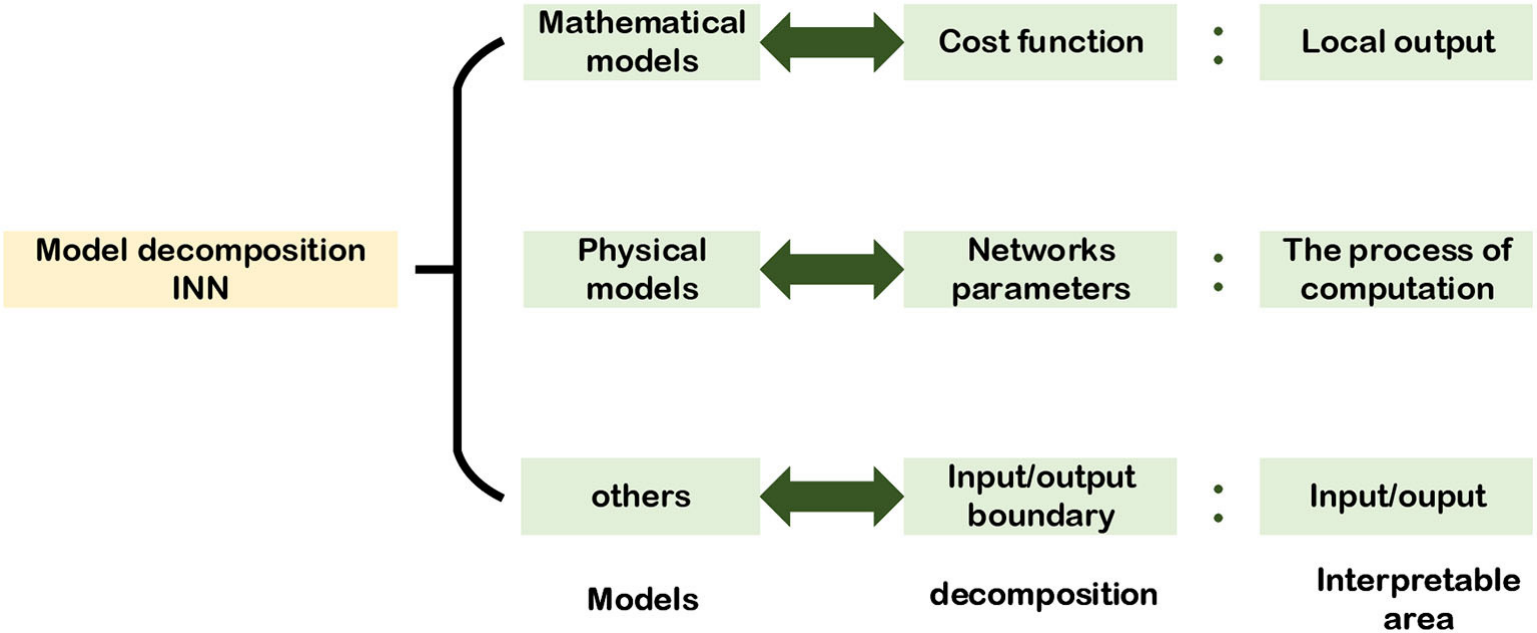
Model decomposition alternative INN.

In this section, we will analyze the way to use mathematical, physical, and other models of a given task to achieve model decomposition alternative INNs from the perspective of different models. Figure 3, presents the alternative approaches of implementing model decomposition alternative INNs and the interpretable regions of the NNs.

### 2.1. Mathematical model-decomposition INN

First of all, the mathematical model has a very broad concept. Almost all problems can be represented by a mathematical model. Without losing the generality, the mathematical model in this article can be expressed by the function *f*(*x*, θ), where *x* represents the input variables, θ represents the parameters of the mapping relationship of the function *f*(*x*, θ). Subsequently, the process of network training is the process of recovering the parameters of the mapping, holds that


(1) $$\hat{y}=f(x_{1},x_{2},\ldots ,x_{i},\ldots ,x_{n};\theta_{1},\theta_{2},\ldots ,\theta_{j},\ldots ,\theta_{m}),$$

where the ŷ denotes the estimated output of the mathematical model. The specific expression of the function *f*(*x*, θ) is not our major concern here. It is decomposed as the optimized objective of the INN for training. The next step is to define the optimized objective or loss function according to the mapping function *f*. Generally, the loss function is denoted by *L*(ŷ, *y*), as shown in Equation (2).


(2) $$L(\hat{y},y)=Distance(\hat{y}-y),$$

where *y* represents the true value of the solution, and *L*(ŷ, *y*) refers to the “distance” between the true value and the model output. In classification fields, “distance” can be expressed in terms of probability, that is, they choose the cross-entropy loss as the loss function. In regression tasks, “distance” is usually expressed in terms of norms, and *l*_1_-norm and *l*_2_-norm are both common choices. In image processing, “distance” reflects the reconstruction performance between the real image and the processed image, and the structural similarity index method (SSIM) is usually used as the evaluation standard for images. Accordingly, it's essential to choose the most suitable loss function when dealing with different types of problems.

**Figure 4:**
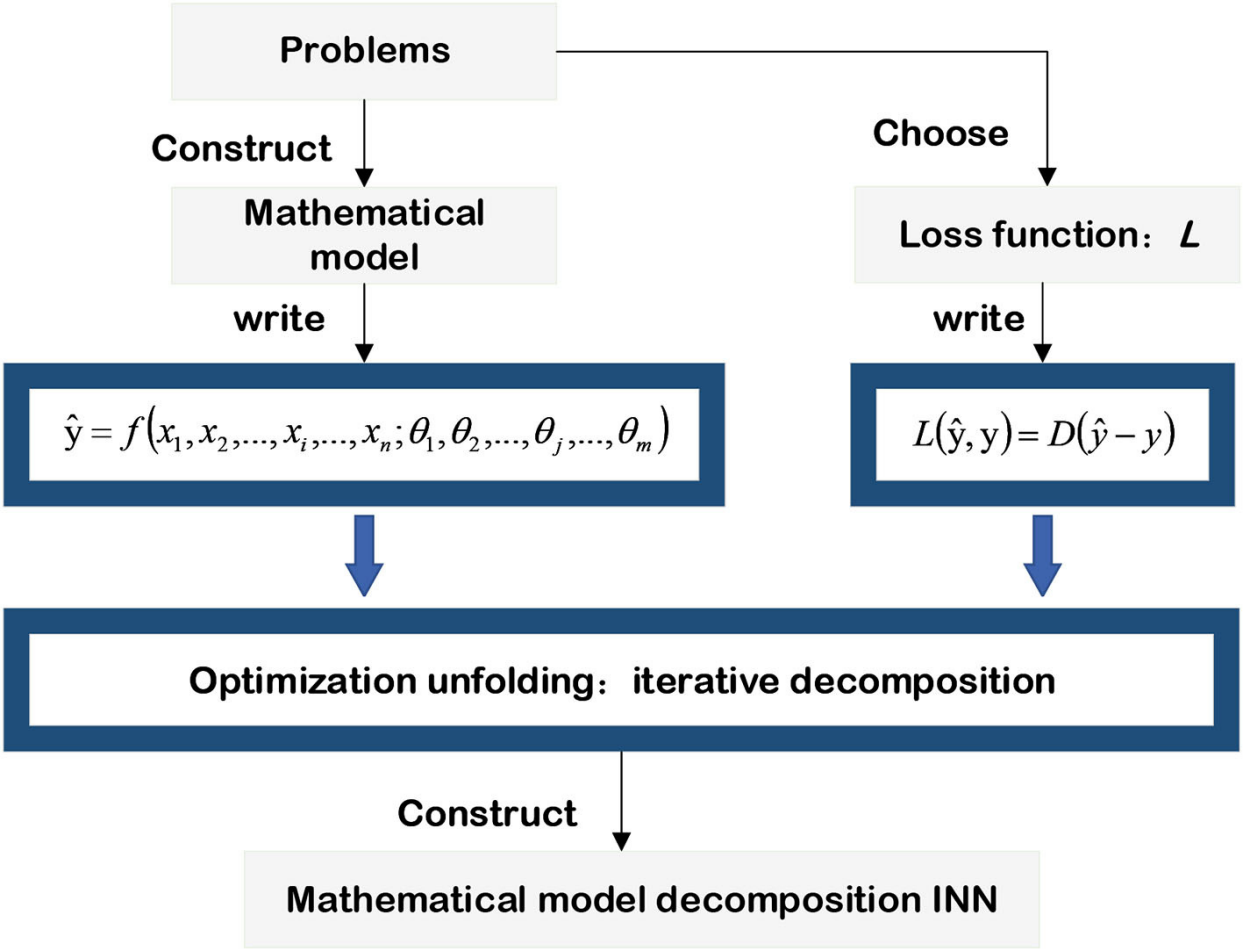
Mathematical model-decomposition INN.

The last step of the INN based on the mathematical model is to decompose the optimized objective, and the alternating direction method of multiplier (ADMM) (Boyd et al., 2011), half-quadratic splitting (HQS) (Wang et al., 2008), and conjugate gradient (CG) (Liu and Storey, 1991; Hager and Zhang, 2006) are widely used in convex optimization problems. In addition, the Markov chain Monte Carlo (MCMC) method (Geyer, 1992; Pereyra et al., 2020) combined with Bayesian estimation is applied to solve non-convex optimization problems. This subsection starts with the regression problem of solving PDE and the image processing problem of image deblurring. It then expands the basic principle of INN based on a mathematical model and gives its general pipeline in Figure 4.

#### 2.1.1. Universal partial differential equations

The mesh-based techniques are widely used in solving differential equations. The basic idea of it is to mesh the differential equations into grids according to the small amount Δ*t* of each step, and then the relationship *f*(*t*_*k*_; *t*_*k*+1_) between the previous moment and the next moment is written with the unit small amount Δ*t*. Finally, by using iterative processes, the relationship between the start time and the end time will be found. However, due to the exponential growth in the number of mesh points with the number of dimensions, it is impossible to solve high-dimensional PDEs with mesh-based techniques. In contrast, the data-driven approach of machine learning (ML) can be more flexible and allows one to drop the simplifying assumptions that are needed to derive theoretical models from the data. Therefore, many scholars consider using ML to solve differential equations, and Rudy et al. (2017) found the terms of the controlling PDE that most properly described the data from a wide library of probable candidate functions using data-driven sparse regression techniques. The basic idea is to use the value of *f*(*x, y*) at a space-time sampling grid to infer the PDE which is satisfied by the system. First, they assume that the PDE can be represented by a series of functions:


(3) $$\begin{array}{l} f_{t}=N\left(f,f_{x},f_{xx},...,x,\mu \right), \end{array}$$

where the subscript represents the differential of the function *f* in time or space, *N*(·) is the uncertain parts in PDEs, and μ represents other parameters that may be related to configuration. Rudy et al. (2017) replace the combination of multiple functions with *F*, and replace other influencing parameters with *P*. Then, the PDE of this system can be written as:


(4) $$\begin{array}{l} F_{t}=\Theta \left(F,P\right)\xi . \end{array}$$

The dictionary Θ contains all possible entries in the PDE for a given system. ξ is a sparse vector, and each non-zero item of it indicates that there is a corresponding entry of the dictionary Θ in the controlled PDE of the system. Each entry of *F* is a specific candidate term for a certain point in space at a certain moment, and each entry of *P* represents the influenced input of the system, which is also assigned to each point and moment. Using sparse regression to find the controlled PDE of a given system without searching for all possible components can effectively reduce the calculation complexity. However, there are still problems with a large number of matrix calculations and the lack of scientific principles in data-driven models. In Rackauckas et al. (2020), Rackauckas proposed an ML method that combines domain scientific knowledge and called this combined model the universal differential equation (UDE). The scientific knowledge is incorporated into the NNs while achieving two goals: reducing the size of the NN structure and speeding up the solution of differential equations.

Let's consider a quadratic ordinary differential equation (ODE) problem. Assuming that there is a natural ecosystem that consists of prey and predators. The variation of prey ẋ is related to its birth rate and capture probability, while the increase of predators ẏ is also related to its birth rate and capture influence. In particular, the capture effect of the prey and predators is mutual, and the variance of both prey and predators can be written as an ODE, namely


(5) $$\dot{x}=ax-bxy,$$

(6) $$\dot{y}=cy-dxy,$$

where *a* and *c* are the birth rates of the prey *x* and predators *y*, and the *b* and *d* are the mutual influence rate of the prey and predator, respectively. In this case, the mutual influence rates of both targets need to be estimated. To address this problem in a standard method, it is necessary to mesh *y* and *x* into data points and then extract the correlation coefficients *b* and *d* by data fitting. Finally, according to the initial ODE, the value of data points at the next moment can be deduced as follows:


(7) $$\begin{array}{l} \frac{x_{k}-x_{k-1}}{t}=ax_{k-1}-bx_{k-1}y_{k-1}, \end{array}$$

(8) $$\begin{array}{l} \frac{y_{k}-y_{k-1}}{t}=cy_{k-1}-dx_{k}y_{k-1}, \end{array}$$

(9) $$\begin{array}{l} x_{k}=\left(at+1\right)x_{k-1}-bty_{k-1}x_{k-1}, \end{array}$$

(10) $$\begin{array}{l} y_{k}=\left(ct+1\right)y_{k-1}-dtx_{k}y_{k-1}. \end{array}$$

The traditional method for deriving the solutions of ODEs is only suitable for the case of low order and low dimension. In the mathematical model-decomposition INN, DL approaches are used to learn unknown interactions between *x* and *y*, which means that the second parts in Equations (5, 6) correspond to NNs.

**Figure 5:**
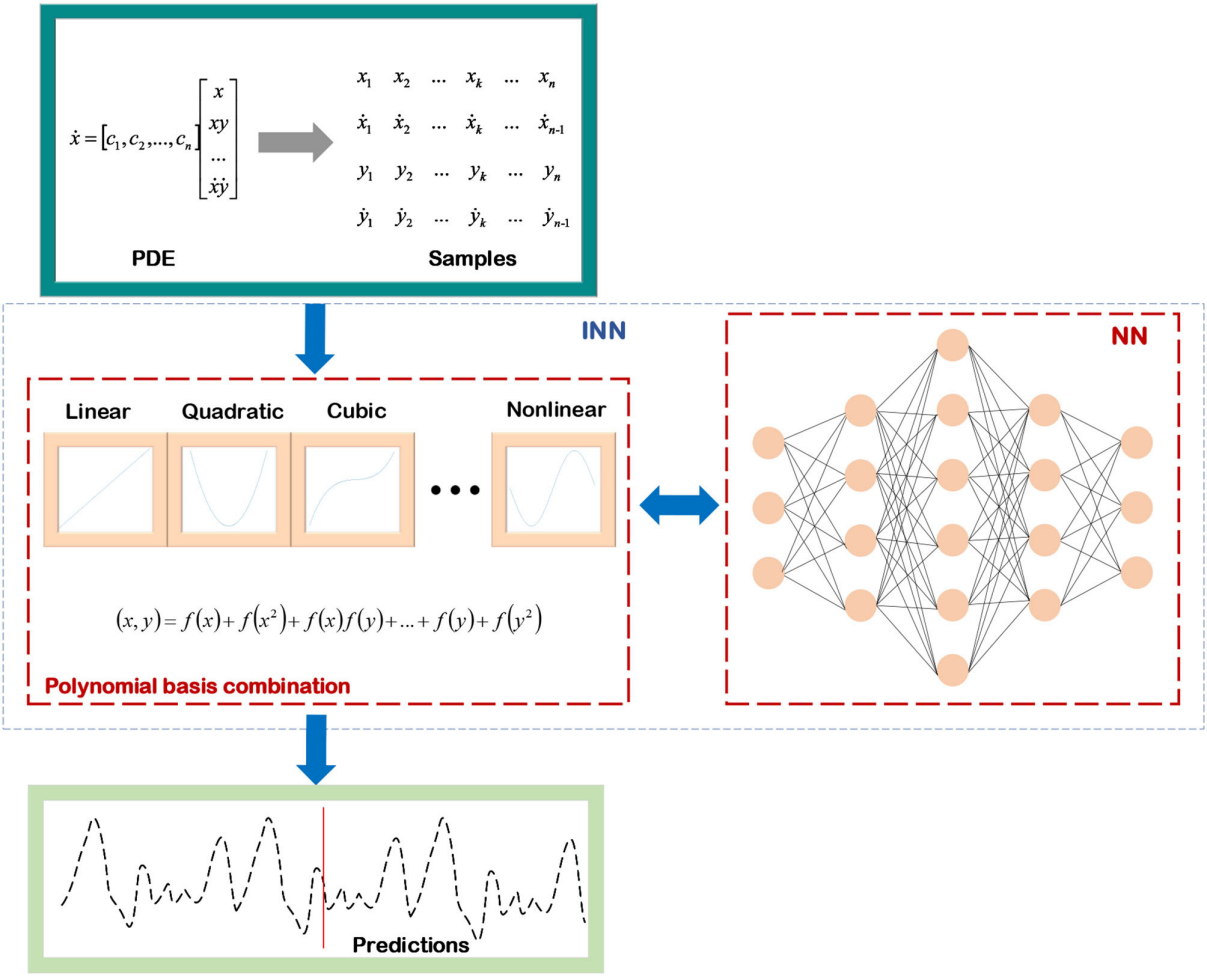
The pipeline of mathematical model-decomposition INN solving UDE.

As shown in Figure 5, combined with the iterative processes for solving ODEs, a universal ordinary differential equation (UODE)-based symbolic regression is constructed, and scientific knowledge and prior conditions are integrated into the process of discovering and solving ODEs, which reduces the number of trials and errors of the network (Li et al., 2020). And due to the prior conditions of a realistic system, it is no longer essential to construct a dictionary matrix containing each derivative term of the independent variable when applying a UODE-based symbolic regression to discover and solve ODEs, and only the finite term polynomial coefficients need to be estimated. Therefore, using the mathematical model-decomposition INN can greatly reduce its computational complexity.

#### 2.1.2. Image deblurring

In the area of image processing, traditional model-based algorithms include image erosion and expansion, edge gradient extraction, Fourier transform, wavelet transform, and matched filtering (Ramella and Sanniti di Baja, 2007; Danielyan et al., 2011; Burger et al., 2012). Due to the domain transformation and matrix inversion procedures in traditional image processing, the edge shadow and ringing effect may happen in the image deblurring. Then, the DL approach has become popular over time, and many researchers employ ResNet for target recognition, UNet for image segmentation, VGGNet for target detection, and generative adversarial network (GAN) for image deblurring (Nah et al., 2017; Kupyn et al., 2018; Tao et al., 2018). However, there are still difficulties for DNNs in realizing image processing with a small sample size, which motivates the development of INNs. These three types of methods and their advantages and disadvantages are compared in Table 1. This subsection mainly introduces how to build an INN for single-image deblurring.

**Table 1:** Companion of various image deblurring methods.

**Methods**	**Advantages**	**Disadvantages**
Matched filters	It can be used to process pictures of unknown blurred kernels with interpretability.	Matched filters require a transform domain, potentially causing ringing and loss of resolution.
Purely DL	It's efficient in real-time with low computational complexity.	DL cannot handle images of types of images that are unknown in the training set.
INN	It can reconstruct pictures of unknown blurred kernels with high efficiency, interpretability, and low computational complexity.	Designing and training INN are complicated techniques.

According to the flowchart given in Figure 4, the first step in realizing a mathematical model-decomposition INN for image processing is to establish a general mapping function. Combined with the properties of the image degradation model, it is assumed that the mapping function can be written as


(11) $$\begin{array}{l} y=Wx, \end{array}$$

where *W* represents the blurred kernel, *x* is the original image, and *y* is the blurred image, which is also the input of the INN. It's defined as an inverse problem, and the final output is the recovered *x*. Then, by adopting the optimization algorithm of the iterative shrinkage and thresholding algorithm (ISTA), the objective function can be expressed as:


(12) $$\begin{array}{l} x=\underset{x}{\mathrm{argmin}}||y-Wx|{|}_{2}^{2}+\lambda ||x|{|}_{1}, \end{array}$$

where λ is the regularization parameter, which is used to ensure the sparsity of the results. According to the ISTA, the optimization problem of Equation (12) can be transformed into iterative solutions, where each iteration computes one step of *x*. Then *x* can be estimated as Beck and Teboulle (2009)


(13) $$\begin{array}{l} x_{k+1}=S_{\lambda }\left(x_{k}-2t_{k}W^{T}\left(Wx_{k}-y\right)\right), \end{array}$$

where *S*_λ_ is a shrinkage operator that updates *x* by performing a soft threshold operation on the outputs. The update formula is as follows


(14) $$\begin{array}{l} S_{\lambda }=\mathrm{sign}\left(x\right)\cdot \max\left\{|x|-\lambda ,0\right\}. \end{array}$$

In order to better represent the updated *x* in each iteration, Beck and Teboulle (2009) separate the input variable *y* and output variable *x* of each iteration in Equation (13), and the suitable step size of gradient descent is replaced by μ_*k*_ = 1/2*t*_*k*_. Then, it can be recast as


(15) $$\begin{array}{l} x_{k+1}=S_{\lambda }\left\{\left(1-\frac{1}{\mu_{k}}W^{T}W\right)x_{k}+\frac{1}{\mu_{k}}W^{T}y\right\}. \end{array}$$

**Figure 6:**
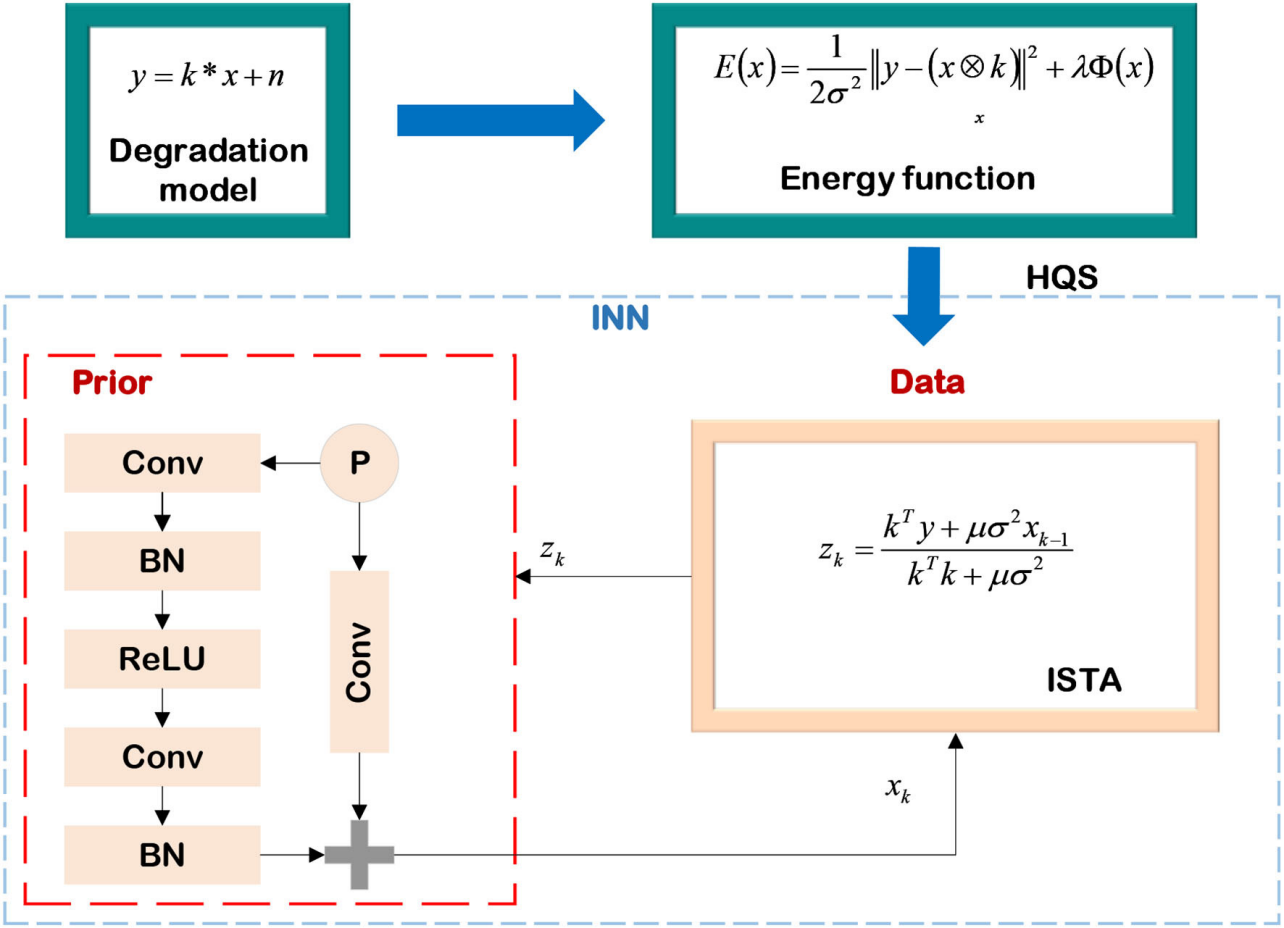
Single image super-resolution based on INN.

Based on the Equation (15), (Li et al., 2020) proposed to unfold the iterative processes into the learnable module. They transfer the parameters in the iteration to the network at the same time and use the method of minimizing the training loss function to continuously estimate the NN's parameters to achieve adjusting the network structure. At this point, the mathematical model-decomposition INNs require fewer iterations than the model-based method, the parameters calculated in the NN have mathematical meanings, and the training of INNs no longer relies on large-scale datasets. Zhang et al. (2020) proposed an INN based on the image degradation model to achieve single image super-resolution (SISR) (Daubechies et al., 2004), the specific process is shown in Figure 6. First, the image degradation as shown in Equation (16) is constructed.


(16) $$\begin{array}{l} y=k*x+n, \end{array}$$

where *k* is the kernel, *x* is the sharp image, *y* is the blurred image, and *n* is the additive white Gaussian noise (AWGN). In order to achieve the SISR, the energy function in the form of Equation (12) is constructed. The goal of deblurring is to minimize the energy function, and its expression is as follows:


(17) $$\begin{array}{l} E\left(x\right)=\frac{1}{2\sigma^{2}}||y-\left(k*x\right)|{|}^{2}+\lambda \Phi \left(x\right), \end{array}$$

where λ is used to control the weight of the prior term Φ(*x*) on the deblurring process, and σ represents the noise coefficient. They use HQS to separate the prior term and the data term of Equation (17), and apply the ISTA method aforementioned to complete SISR. The decoupled data term and prior term are expressed as Monga et al. (2021)


(18) $$\begin{array}{l} z_{k}=\arg\underset{z}{\min}||y-\left(k*z\right)|{|}^{2}+\mu \sigma^{2}||z-x_{k-1}|{|}^{2}, \end{array}$$

(19) $$\begin{array}{l} x_{k}=\arg\underset{x}{\min}\frac{\mu }{2}||z_{k}-x|{|}^{2}+\lambda \Phi \left(x\right), \end{array}$$

where μ is the step size.

**Figure 7:**
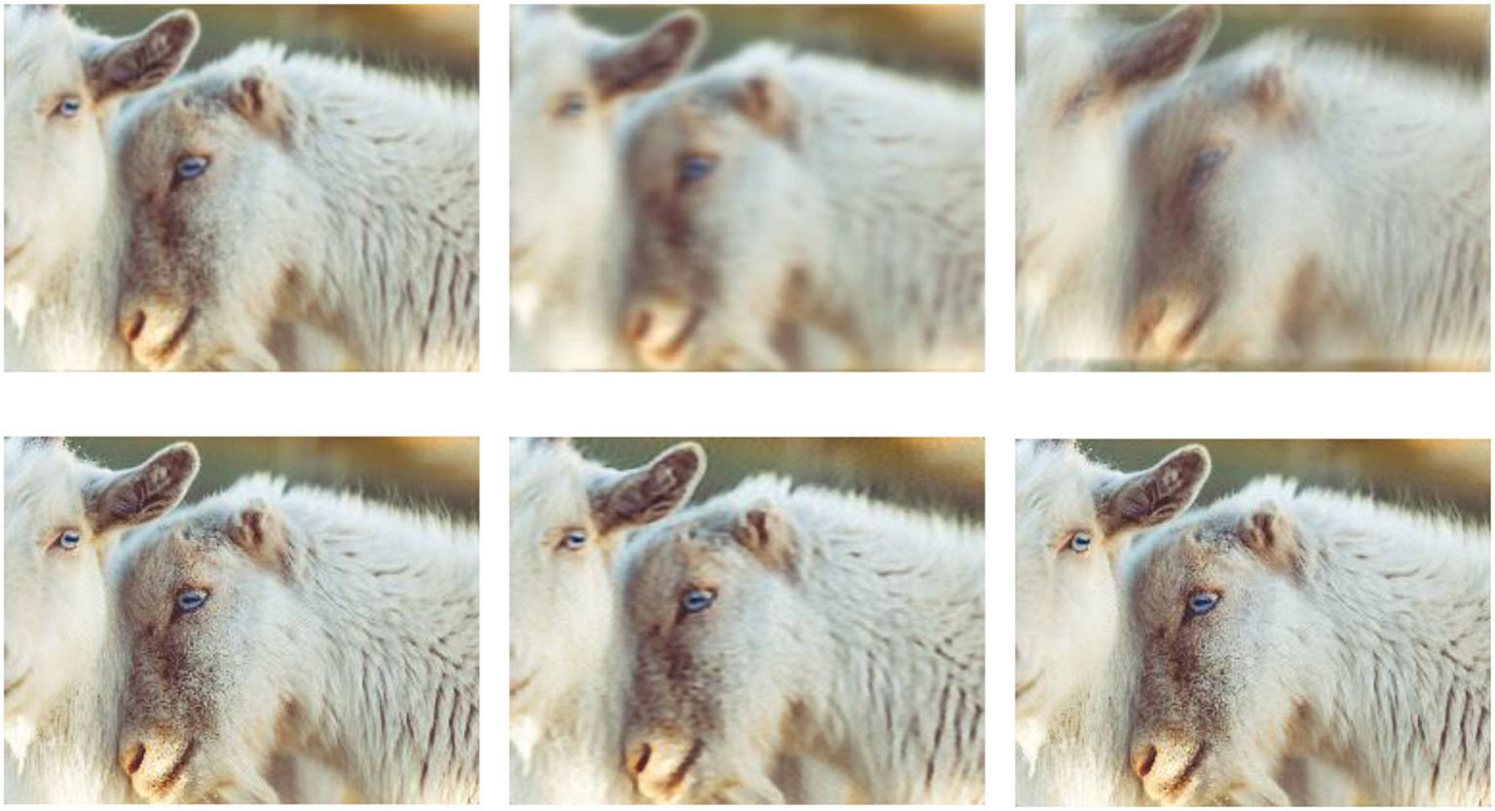
Examples of SISR with different blurred kernels. The first row includes three low-resolution images, and the images in the second row correspond to the super-resolution results of the low-resolution images in the first row. The images from left to right are distinguished by different blurred kernels, which are homogeneous Gaussian kernels, anisotropic Gaussian kernels, and motion-blurred kernels, respectively.

The pseudo-inverse algorithm is used to directly calculate the value of *z* in the data part. Nevertheless, the pseudo-inverse bias matrix is a large filter kernel, which can be solved by cascading into multiple smaller kernels. For the SISR problems, the prior term is considered an image denoising process of *z*, which can be replaced by the form of a ResNet. Following the method given in Daubechies et al. (2004), this subsection implements the deblurring on the DIV2K dataset (Timofte et al., 2018), and Figure 7 shows the deblurring results of the INN when the images are degraded with different blurred kernels.

### 2.2. Physical model-decomposition INN

**Figure 8:**
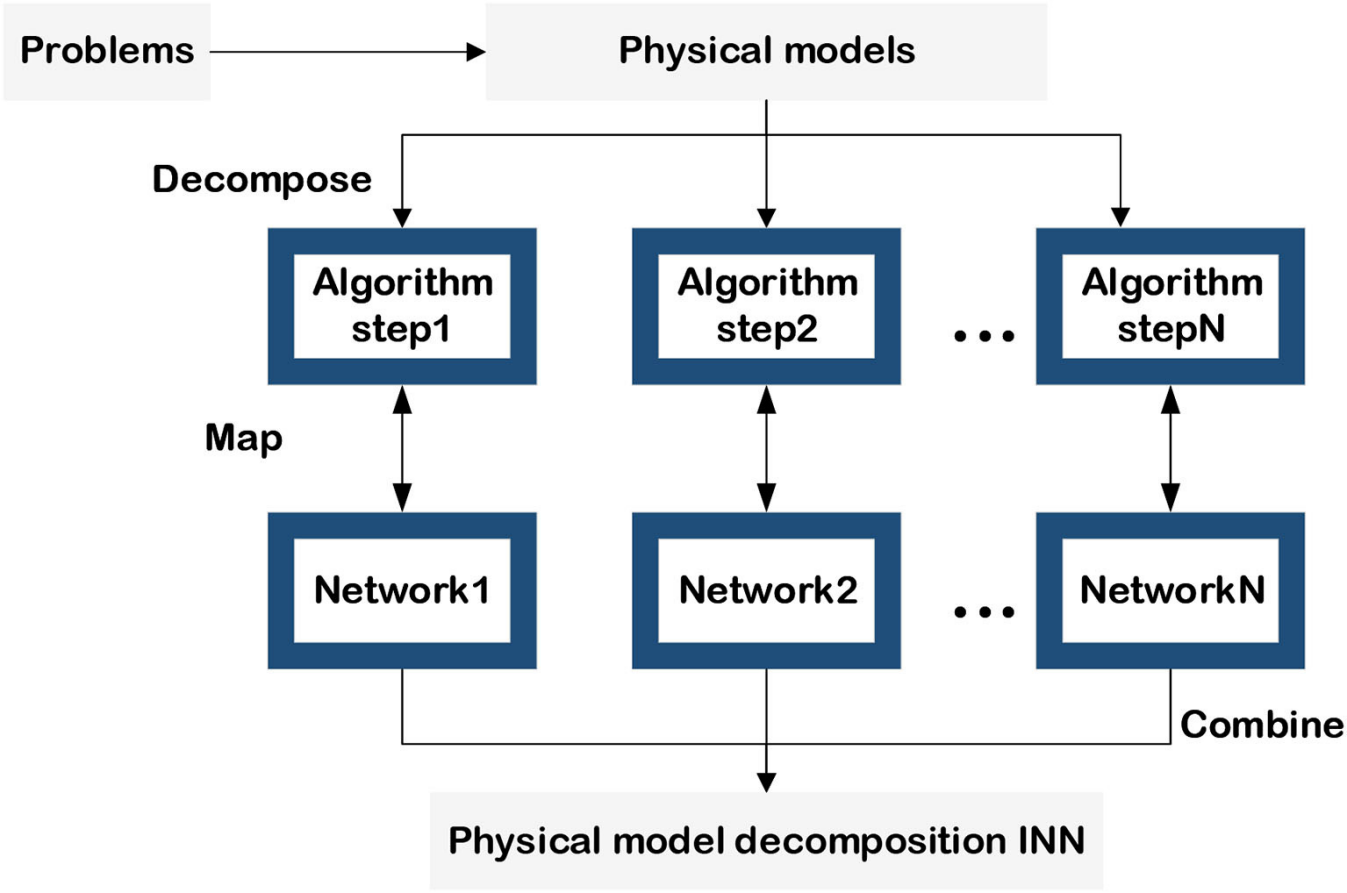
Physical model-decomposition INN.

Physical model-decomposition INNs are primarily concerned with issues in physical electromagnetism and dynamics. Actually, the term “model” here not only refers to physical models described by a formula but also includes constraints and principles in physics. Compared with the mathematical model-decomposition INNs, the basic idea of this approach is to convert the domain knowledge contained in the physical model into NN parameters and replace the calculation processes in the physical model with the layers of the NNs, as shown in Figure 8. Starting with an electromagnetic model, the tasks of wave dynamics prediction and forward and inverse scattering predictions are described in detail and the turbulent motion prediction is introduced briefly.

#### 2.2.1. Wave equation prediction

**Figure 9:**
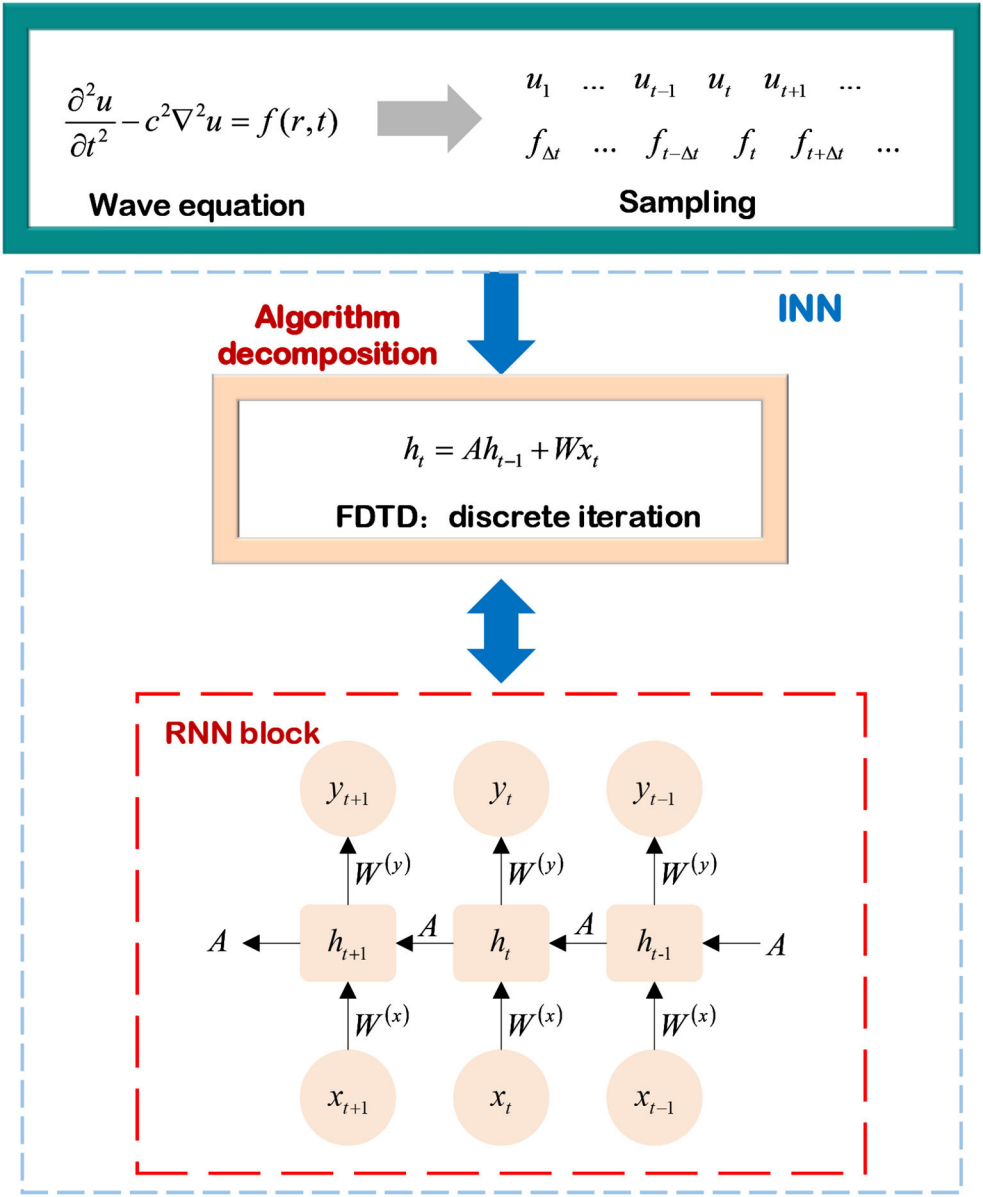
Wave equation prediction based on INN.

The electromagnetic wave radiates outward with a specific pattern in free space, and its fluctuation mode is determined by the exciting source and medium characteristics. The propagating direction and fluctuation state at each point in the wave propagation are related to the previous moment, implying that wave propagation is the same as the continuous time series. This subsection introduces a continuous physical model for wave propagation in free space. Assuming that the exciting source *f*(*r, t*) emits spherical waves, and Equation (20) shows the time domain wave-based dynamics of the scalar electric field *u* in free space.


(20) $$\begin{array}{l} \frac{\partial^{2}u}{\partial t^{2}}-c^{2}\nabla^{2}u=f\left(r,t\right), \end{array}$$

where *c* and *t* represent the speed of light and time steps, respectively. Compared with solving PDEs with mathematical model-decomposition INN, the formula (20) is discretized by finite difference to obtain the form of the wave equation related to the temporal step Δ*t*, as formulated:


(21) $$\begin{array}{l} \frac{u_{t+1}-2u_{t}+u_{t-1}}{\Delta t^{2}}-c^{2}\nabla^{2}u_{t}=f_{t}\left(r,t\right), \end{array}$$

where the subscript *t* is the value of a scale electric field at the given time. Fan et al. (2021) built a mapping between the physical parameters in the discrete wave equation and the neurons in the RNN (Hughes et al., 2019), as shown in Figure 9.

In an iterative process, the state vector *h*_*t*_ is defined as the combination of scalar field's values within a temporal step Δ*t*, which is a column vector connected to sampling time and can be expressed as Equation (22). Then, they substitute it into Equation (21) to obtain the state vector of the scalar field, as shown:


(22) $$h_{t}=\left[\begin{array}{c} u_{t+1}\\ u_{t} \end{array}\right],$$

(23) $$h_{t}=\left[\begin{array}{cc} 2+\Delta t^{2}c^{2}\nabla^{2} & -1\\ 1 & 0 \end{array}\right]h_{t-1}+\Delta t^{2}\left[\begin{array}{c} f_{t}(r,t)\\ 0 \end{array}\right].$$

Considering the wave equation prediction, the process of calculating the state vector of the scalar electric field is converted into a layer of RNN. Then, the hierarchical model of the RNN can be written as


(24) $$\begin{array}{l} h_{t}=\sigma^{\left(h\right)}\left(W^{\left(h\right)}\cdot h_{t-1}+W^{\left(x\right)}\cdot x_{t}\right), \end{array}$$

(25) $$\begin{array}{l} y_{t}=\sigma^{\left(y\right)}\left(W^{\left(y\right)}\cdot h_{t}\right), \end{array}$$

where *W*^(*h*)^, *W*^(*x*)^, *W*^(*y*)^ are the trainable parameters in the RNN, σ^(*h*)^, σ^(*y*)^ are nonlinear activation functions. Combining Equations (23, 24), the mapping between the physical parameters of the discrete wave equation and the weight parameters of the RNN state equation can be constructed. They are shown as:


(26) $$\begin{array}{l} W^{\left(h\right)}=\left[\begin{array}{cc} 2+\Delta t^{2}c^{2}\nabla^{2} & -1\\ 1 & 0 \end{array}\right], \end{array}$$

(27) $$\begin{array}{l} W^{\left(x\right)}=\Delta t^{2}. \end{array}$$

The input parameters *x*_*t*_ of the wave-based RNN are determined by the exciting source *f*_*t*_(*r, t*) in free space, and the trainable weights of the RNN are related to the speed and scope of wave propagation. This means that the structure of the RNN is reasonably mapped to a physical model, and the parameters of the NNs have clear physical meanings. The wave-based RNN combined with FDTD to achieve wave equation prediction has certain interpretability. Furthermore, assuming that there is a medium in space, it's achievable to obtain the mode of wave propagation in the medium with the backward of the state matrix and estimate the dielectric constant of the dielectric layer and some other dielectric parameters from the correspondence between the trainable weight matrix *W*(*h*) and the propagation velocity.

#### 2.2.2. Electromagnetic scattered field estimation

The numerical calculation method for the electromagnetic scattering problem can be adaptive to estimate the scattering field of various shapes of the medium, but it is faced with obstacles of high computation complexity in complicated models. Using the DL method to accelerate the numerical calculation of electromagnetic scattering problems is a new direction in this field, which combines the parallel computing ability and high efficiency of NNs with the generalization ability and stability of numerical calculation methods. The concept of this type of INN accelerating numerical calculation can help us address more electromagnetic scattering issues in the future.

**Figure 10:**
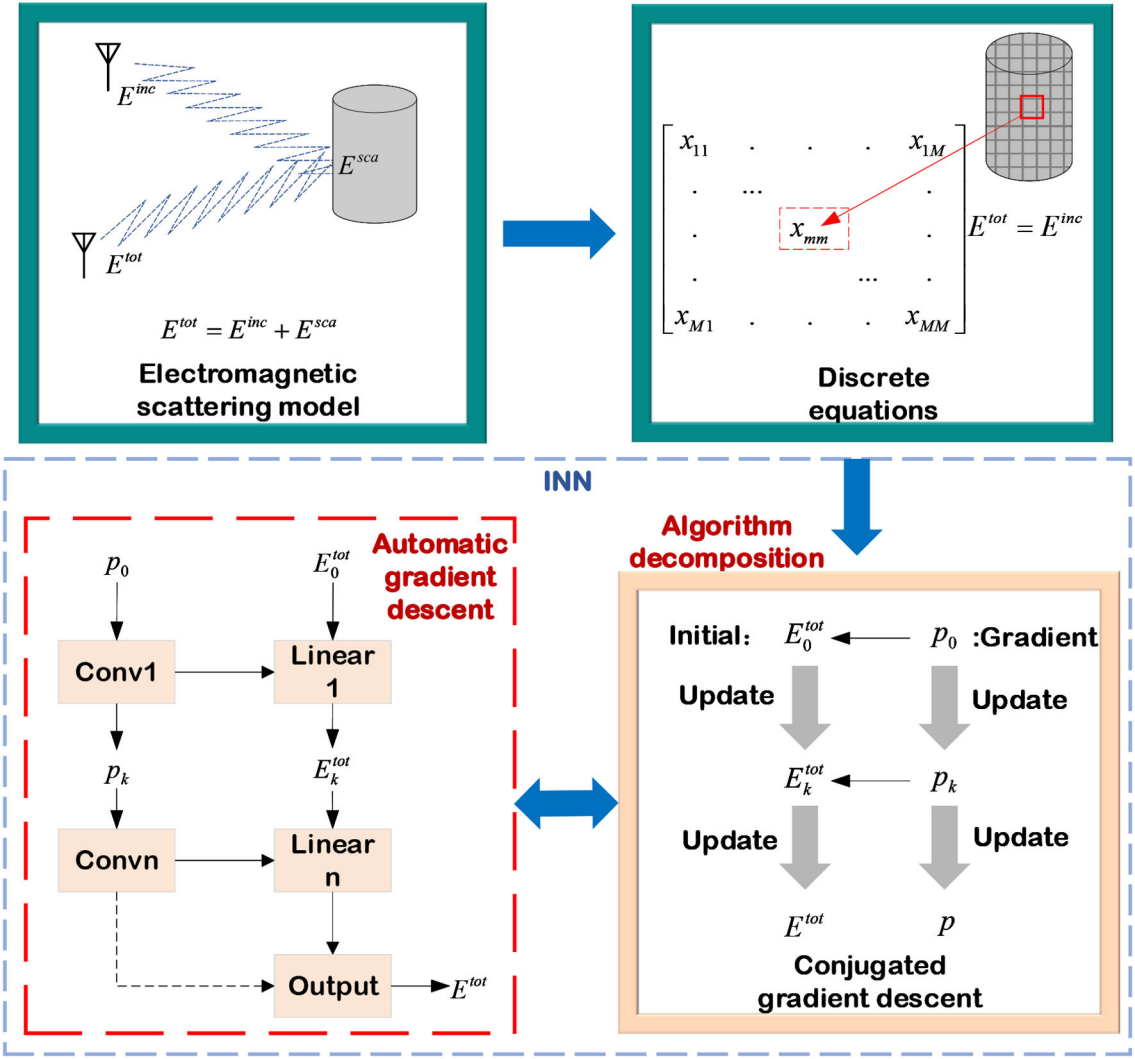
INN accelerating electromagnetic calculation.

This subsection mainly discusses how to use physical model decomposition INNs to solve the forward scattering problem of dielectric layers as shown in Figure 10. Starting with converting the continuous forward scattering equation into a discrete scattered field model and then combining it with the conventional electromagnetic calculation algorithm to solve the discrete scattered field problem, it is ultimately replaced with a NN.

Considering a lossy dielectric scatterer in two-dimensional free space. The position of this scatterer is denoted as *r* = (*r*_*x*_, *r*_*y*_). It is assumed that the electromagnetic wave emitted by the transmitting antenna is a transverse electromagnetic wave in the *z* direction. Then the permeability of the scatterer remains consistent with free space, and the complex permittivity varies with distance and frequency, which can be expressed as


(28) $$\begin{array}{l} \varepsilon \left(r\right)=\varepsilon_{0}\varepsilon_{r}\left(r\right)-\frac{j\sigma \left(r\right)}{\omega }, \end{array}$$

where ε_0_ is the permittivity in free space, ε_*r*_ is the relative permittivity, σ is the conductivity, and ω is the angular frequency of the incident electromagnetic wave. For any direction incident field *E*^*inc*^(*r*), the computation principle of scattering in the far field is consistent with the electric field integral equation (EFIE). Therefore, the total electric field *E*^*tot*^(*r*) can be measured with the incident electric field plus the scattered electric field obtained by the secondary radiation on the surface of the scatterer, as shown in (29):


(29) $$E^{tot}(r)=E^{inc}(r)+k_{b}^{2}\int_{D}G\left(r-r'\right)\chi \left(r'\right)E^{tot}\left(r'\right)dr',$$

where *k*_*b*_ represents the wave number. In the two-dimensional case, Green's function of free space in cylindrical coordinates is denoted by *G*(*r*−*r*′). The contrast of permittivity is χ(*r*) and *E*^*sca*^(*r*^*R*^) is the scattered field at the distance *r*^*R*^. The scattered electric field can be regarded as the secondary radiation of the induced current *J*, which can be written as


(30) $$\begin{array}{l} J\left(r\right)=\chi \left(r\right)E^{tot}\left(r\right). \end{array}$$

In this subsection, the impulse function is used as the basis function, and the discretized matrix equation is constructed by the point test function. The scattered region *D* is divided into *M* sub-regions, and in the *m*-th subregion, its EFIE can be written as


(31) $$E_{m}^{tot}+\frac{j}{4}k_{b}^{2}\sum_{s=1}^{M}\chi E_{s}^{t,ot}\int_{D_{S}}H_{0}^{\left(2\right)}\left(k_{b}\left|r_{m}-r_{s}^{\prime }\right|\right)dr_{s}^{\prime }=E_{m}^{inc}.$$

Then the matrix equation for all regions of interest can be formulated as


(32) $$\begin{array}{l} \left(I+Z\chi \right)E^{tot}=E^{inc}. \end{array}$$

The conjugate CG, which is a hybrid of the steepest descent algorithm and the Newton iterative approach, is used to solve the EFIE problem. Moreover, it is also one of the most efficient algorithms for addressing nonlinear optimization problems and solving sparse systems of linear equations. The CG method was first proposed by Hestenes and Stiefel (1952), and its essential point is that in the computation process, each search direction is conjugated to each other, and these search directions are calculated by the negative gradient and the search direction in the previous step. Wei and Chen (2019) replaced the process of updating the gradient direction and the total electric field *E*^*tot*^ with two cascaded NNs, respectively. In the first replacement, NN is used to predict the gradient direction of the next stage, and the step size and weight automatically assigned by the network are used for updating. The input of the network includes the residuals of the previous two moments, denoted by *pr*_*k*_ and *r*_*k*−1_, and the gradient direction of the previous moment, denoted by *p*_*k*_. Then, the process of updating the gradient direction can be consequently expressed as


(33) $$\begin{array}{l} p_{k+1}=f\left(p_{k},r_{k},r_{k-1},\theta_{k}^{p}\right), \end{array}$$

where $\theta_{k}^{p}$ is the weight in NNs. Similarly, the process of computing the total electric field based on the CG algorithm is replaced with several cascaded NNs, while the step size and weights are automatically updated by NNs' back-propagation. The process of finally computing the total electric field *E*^*tot*^ is shown as:


(34) $$\begin{array}{l} E_{k+1}^{tot}=E_{k}^{tot}+f_{p}\left(p_{k},r_{k},r_{k-1},\theta_{k}^{p}\right), \end{array}$$

where *f*_*p*_(·) is the optimized NN of the total electric field. The NN replaces the standard gradient descent approach in the forward scattered field computation. In conventional electromagnetic calculation methods, each iterative update requires calculations of the gradient direction and selections of step size. Only by selecting the appropriate step size, the forward scattered field can be estimated quickly and accurately. In comparison to the pure data-driven technique, the whole network structure of theINN incorporates some of the theoretical information. As a result, it does not need a large amount of data to predict the mapping function, allowing the capacity to minimize data dependency.

#### 2.2.3. Turbulent motion prediction

Predicting the direction and speed of turbulence has crucial uses. The motion of plasma turbulence, for example, can interfere with satellite operations and space communications in interplanetary space. The movement of atmospheric turbulence in the atmosphere influences the trajectories of tornadoes, tsunamis, and cold waves. Therefore, being able to accurately predict the trajectory of turbulence is one of the most essential research in the field of fluid dynamics. According to the principle of Figure 3, turbulence motion prediction can be regarded as a task of solving PDEs. In the process of constructing PDEs, some physical constraints are drawn into account to control the multiple-order terms contained in the differential equations. Hence it is possible to solve this prediction by employing an INN that is the same as PDEs. This kind of physical model-decomposition INN for turbulence prediction is finally divided into the following four steps (Kochkov et al., 2021):

1. Constructing differential equations combined with physical constraints.
  According to dynamic principles, the velocity and trajectory of turbulent flow are only related to a few influencing factors. As a result, the general model of turbulence motion is sparse in space and can be composed of finite non-zero terms.
2. Time series discrete sampling
  The constructed turbulence model is discretely sampled according to Δ*t* of each step, and the discrete model solved by the iterative approach can be split into smaller components.
3. Discrete turbulence motion model combined with NNs.
  The PDEs are decomposed into finite terms containing certain parameters and unknown equation terms that are related to known terms.
4. Training and testing the INN.

The decomposition of known and unknown terms from a turbulence model is a critical step in building an INN. It not only helps to reduce unknown parameters in NNs but also confines the convergence of the loss function. Furthermore, the model decomposition affects the ultimate accuracy and the difficulty of network training. The known terms in the turbulence prediction INN guarantee that the final prediction results are comparable in overall trend to the precise solution. Simultaneously, the unknown items related to the known items adjust the model at certain tiny values, allowing the final outcome to satisfy our expectations.

### 2.3. Other model-decomposition INN

There are many challenges in the biochemistry area right now that cannot be properly represented by a mathematical model, yet they nonetheless include a wealth of domain knowledge in processing. In this section, other models are used to generalize such issues, and all the processes of using domain knowledge to modify the input and output of DL are collectively referred to as other model-decomposition INNs. Therefore, these INNs focus on improving the front-end input data or correcting the terminal output results. Its interpretability is mainly realized in data processing and hyper-parameter configuration rather than in NN's layer design. Following the successful training of a “black box” NN, the analysis of interpreting the network structure, results, datasets, and so on is called “*post-hoc* interpretability,” which means that *post-hoc* interpretability does not affect the NN before training. For example, in fluorescence image reconstruction, the probability and shape of the target appearing in a specific area are determined by a theoretical model, and these theoretical models constrain the final output through template matching. In ultrasound imaging, noise is mixed with the input signal. Using PCA to process the ultrasound will greatly improve imaging performance. For a complex value classification network, dealing with the real and imaginary parts of a complex separately cannot reflect the backward of complex values. Redefining the forward calculation and backward propagation of the complex value network makes the NN training more realistic and increases the input information.

## 3. Semantic INN

**Figure 11:**
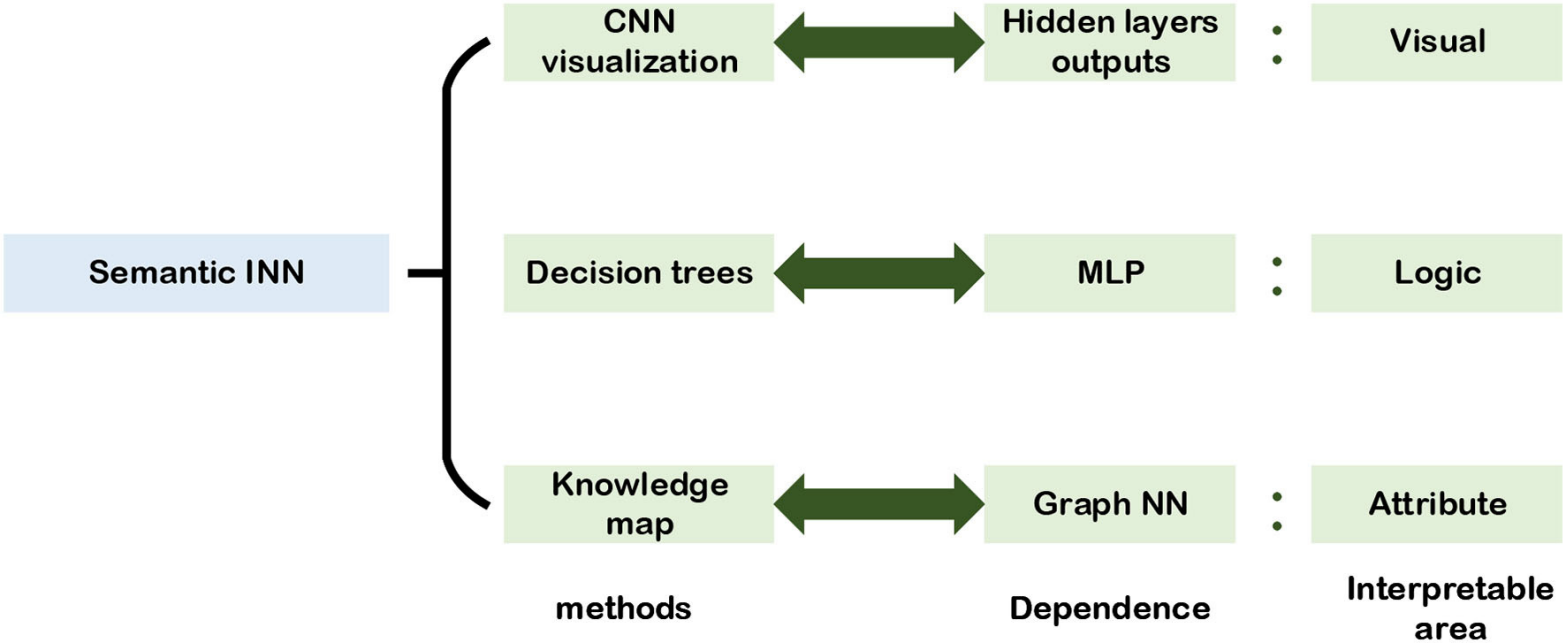
Semantic INN.

Semantic INN is the interpretable analysis method designed for the engineer. Its core concept is to explain the NNs from the standpoint that the engineer enables to see visually, analyze logically, and understand attributes. To this end, the first for the engineer who works on the semantic INN is to evaluate results visually and then analyze reasons logically, which are used to explain the reason in an accessible way. In this section, semantic INN designed for engineers is divided into three aspects which are vision, logic, and attributes, respectively. As shown in Figure 11, it illustrates the semantic INN structure and its interpretable regions based on the three aspects.

Semantic INN starts with the visualization of convolutional layers by plotting the heat map of each layer to reflect changes during network training. Then, combining decision trees and DL methods, logic calculations are drawn into the NNs so that there is certain logic information in the network layers, and explainable trees are extracted from NNs to explain the network structures. At the same time, there are also many studies directly starting from the attribute semantics of the target to build INNs.

### 3.1. Visualization of CNN

From a visual point of view, we hope to see the relationship between each output result of the network during the training process and the input data, especially in the classic image recognition classification problem, and analyze how the network recognizes the target from the input image. This method started with AlexNet visualizing the convolution kernel of the first layer, and then Zeiler and Fergus (2014) proposed a more explicit visualization method to comprehend the visualization results of convolutional layers, which was the pioneering work of visualization research. Moreover, numerous scholars who analyze and understand visualization results in image classification and recognition (Yosinski et al., 2015). The core concept of CNN visualization is to draw all the feature maps of each hidden layer in the CNN and examine the activation values of feature maps in the CNN. Finally, the visualization results are realized by extracting the convolution kernels from the pre-trained network, which is a process of deconvolution.

This section mainly discusses how to inversely map the feature map to the original pixel image, and comprehend the function between the feature map of each layer in CNN and the pixel image. Firstly, the process of convolution calculation in a pixel image can be divided into the following four steps:

1. Convolution kernel
  The process of convolution may be thought of as an operation in the field of image filtering, and the size of the filter is proportional to the size of the convolution kernels.
2. Normalization
  Normalization is an equalization operation on each pixel of the feature map, and not all convolutional layers need to be normalized.
3. Activation function
  The activation function ensures the threshold of each feature map. Usually, the feature map of each layer is a positive value, and the Rectified Linear Unit (ReLU) activation function is widely used.
4. Pooling kernel
  Pooling reduces the size of the feature map in the previous step, which is an irreversible down-sampling process.

The fundamental aim of CNN visualization is to combine the feature maps from layers to analyze the influence on input. The feature map deconvolution procedure is the inverse of the pixel image convolution calculation. To correlate to the convolution, the deconvolution procedure is similarly separated into four steps (Zeiler and Fergus, 2014):

1. Up pooling
  The feature map needs to be up-sampled to the same size as the original image of the previous layer, and the up-sampling method is up-pooling. Its basic idea is to record the position of the maximum activation value of the pooling output in the original image, and then only activate this position, while the other positions are zero.
2. Activation function
  The pixel image still needs to keep the pixel value positive, and the activation function in the deconvolution can be consistent with the activation function in the convolution.
3. Denormalization.
  Denormalization is the equalization processing of the entire picture, which can be omitted or multiplied by a fixed intensity.
4. Deconvolution
  The deconvolution procedure is the core of CNN visualization, and it is also a filter. It can be achieved by multiplying the feature map with the transpose of the convolution kernel.

**Figure 12:**
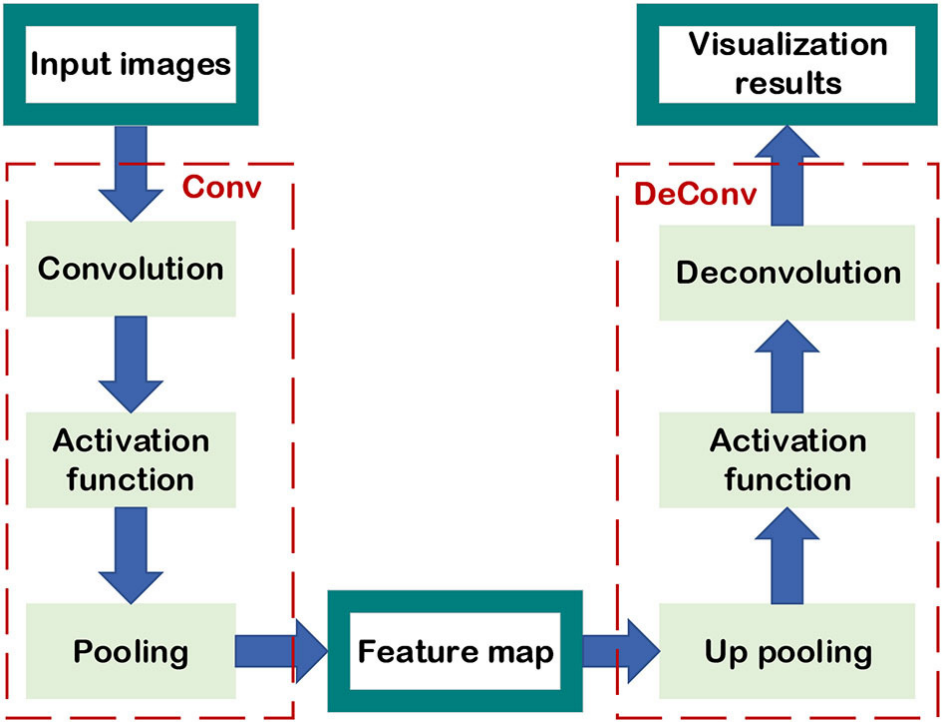
Visualization of CNN.

As shown in Figure 12, the flowchart of performing a convolution operation on a pixel image to obtain a feature map performing a deconvolution operation with the feature map to obtain an approximate pixel image is given. Through the deconvolution procedure, the information of the particular feature map corresponding to an input image can be visualized, which is used to analyze the different functions between low-level layers and high-level layers to extract pixel image characteristics. Yosinski et al. focused on CNN visualization and pointed out the difference in performance between shallow network and deep network in image feature extraction (Yosinski et al., 2015).

The convolution kernels of the lower layers can be drawn by flattening, and they extract the edge, color, and other macro characteristics of the pixel image. The contribution of shallow structures to the image recognition task can be represented by convolution kernel activated values. Meanwhile, the deep convolution kernel can extract more complex characteristics of the pixel image although the function of the deep structure cannot be judged directly from the convolution kernel shapes and the output of the convolution kernels. Hence, using CNN visualization is one of the most suitable methods to map the output of the deep layers' kernels into the original pixel image, which reflects the texture of the pixel image, and the deeper the feature map, the more specific features are extracted. Besides, it's known that convolution is proposed based on the translation and scaling invariance of the image. Owing to the linear transformation of the image, the edge, and color retrieved by the low-level network will change, while the abstract texture extracted by the high layers will not change. Finally, the visualization of CNN can be applied to not only illustrate the operations of the NN layers but also to verify the role of various convolution kernels in accomplishing tasks. It can further modify and improve the initial structure according to the outcomes of feature map visualization and increase the performance of the NN.

Recently, verification and validation (V&V) of NN is well accepted in the autonomous safety assessment. (Huang et al., 2020; Rajabli et al., 2020). The pipeline of the autonomy system is to use physical sensors to provide image information, and perceptrons to provide image interpretation. As clarified in the standards for autonomous systems (ANSI/UL 4600) (Koopman et al., 2019), when verifying the correctness of classifiers, the classification result can only be accepted if it has been obtained with the consistency of human expectations. Furthermore, the autonomy system needs to provide a mapping of the NN input of an ontology of the operational design domain in addition to the classification result. To this end, using the visualization of CNN to achieve V&V in the autonomy system is reasonable, and the NN will provide a transformation of the NN output of different layers to an activating value of the original images. For example, a pre-trained CNN presents the classification result of the autonomous system, and the CNN visualization approach is used to convert the investigated image to the correct ontology member.

### 3.2. Generation and extraction of decision trees

**Figure 13:**
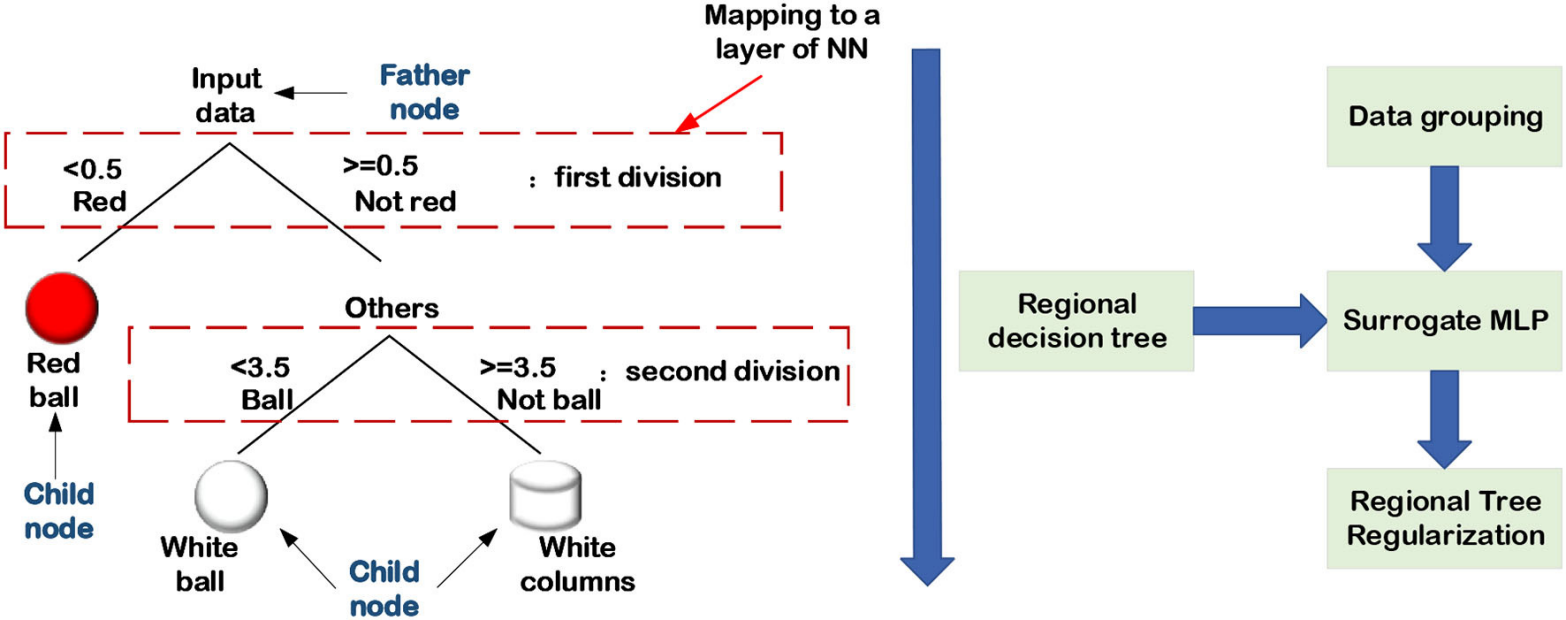
Regional decision trees generation.

**Figure 14:**
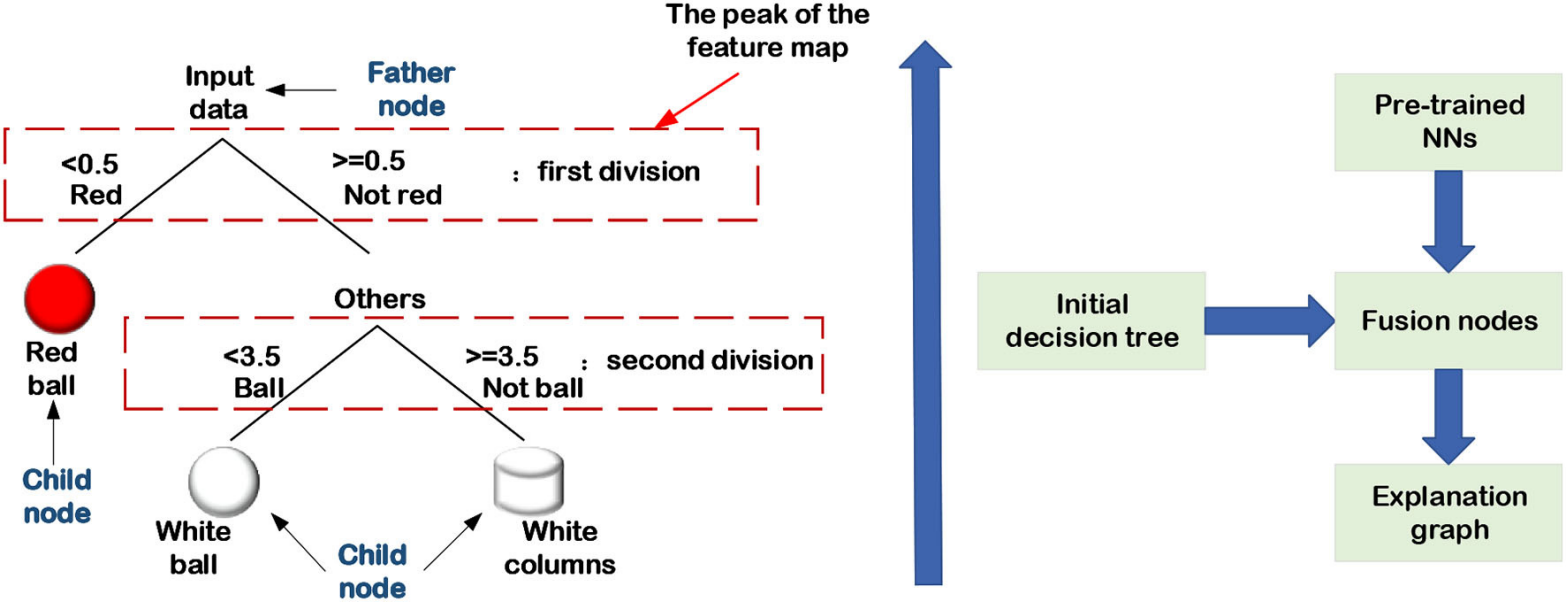
Explanation graph extraction.

From the perspective of human reasoning, it's reasonable to combine the DL methods with decision trees to achieve semantic INN. The decision tree can assist engineers to perform classification tasks by utilizing the meaning of their nodes and edges. In particular, a decision tree contains parent nodes, child nodes, and top-down edges. A parent node can connect to two or more child nodes, and the message transfer on the adjacent edge can only be from the previous parent node to its child nodes. In other words, the decision tree is a top-down structure, which is widely used in classification and regression problems with supervised datasets. For a given dataset, the first step to extracting its decision tree is to encode the labels of targets. For instance, considering a multi-class classification shown in Figures 13, 14, the red color is coded as “0” while the white denotes “1.” The sphere is coded as “3,” and the cylinder is labeled as “4.” Assuming that the decision of the first layer is color, and only the ball is red in the original dataset, the decision from the parent node to the red child node must be a red ball. Keep splitting down until all the decisions are made, and then a standard binary decision tree will be constructed consequently. There are many methods to establish decision trees, and their basic idea is to split nodes from top to bottom, such as CART, ID3, and C4.5 (Charbuty and Abdulazeez, 2021). Because of the logical interpretability of decision trees, it's a reasonable way to combine the DL methods with decision trees to achieve semantic INN, which is the main topic in this section.

The approach of using decision trees to handle data classification and regression issues is consistent with the agent's inductive interpretation and that the decision tree's hierarchical structure is similar to the network layers. Therefore, it is effective to improve the interpretability of NNs by combining the inductive judgment of decision trees. Frosst and Hinton (2017); Wu et al. (2018, 2020) propose a strategy for increasing the interpretability of the trainable network by adding decision tree regularization to the regularized network which can be divided into a global regularization, and a regional regularization network. The objective of adding decision trees is to constrain the network training, and local regularization can better adapt to data changes in data classification issues. The process of combining the decision tree to regularize the network is divided into the following four steps:

1. Data grouping.
  When grouping the input data, the linear segment can be used as a data grouping method.
2. Decision tree extracting.
  The ML method is used to classify each group of data, and construct a decision tree for each group of data.
3. Decision tree regularization network constructing.
  The decision tree is added to the network training as the regularization part of NNs, and the trainable network is constrained by the decision tree.
4. Training a semantic INN.

The decision tree isn't differentiable in step (3), which can't be immediately put into the network training as a regularization function. Hence, it is necessary to construct a map from the decision tree to the trainable structure.

This non-derivable decision process can be achieved by converting it into a layer of a linear transformation, as shown in Figure 13. The strategy adopted by Wu et al. was to convert the decisions in each layer of a decision tree into multi-layer perceptrons (MLPs) (Wu et al., 2018). They use the fully connected layer to realize each round decision, which means that the number of layers in the MLP is consistent with the depth of the decision tree. Then, using a pre-trained network, the decision tree is transformed to the MLP in which the corresponding relationship between the nodes is encoded in the activation values of feature maps. Consider an input image as a parent node, which should be divided into several child nodes at a given level. In the MLP, the comparable procedure is that several feature maps are generated in the input image via a fully connected layer, and these feature maps continue to split downward as child nodes of the subsequent layer. In general, to employ decision tree regularization to achieve semantic INN combined with the logical level of the agent, it is important to define or train a regularization network in advance.

In addition to constructing the map of decision trees to trainable structures as aforementioned, there are scholars who establish the map from NNs to the explanation graph. Sun et al. (2020) demonstrate the way to use statistical fault location (SFL) techniques from software engineering to provide a high-quality interpretation of DNN's output and propose an algorithm and tool called DEEPCOVER. Their method uses SFL to synthesize a ranking of input features and constructs explanations of DNN decisions based on ranking. Zhang et al. (2017, 2019a) presented the bottom-up technique of using an explanation graph in combination with CNN visualization to extract the hidden semantics of pre-trained CNNs. The essential idea of this technique is to consider the activation peak value of each feature map as a child node, and network layer connections as the edges between the child node and the parent node. It consists mostly of the two phases listed below:

1. Initial decision tree
  Before explaining graph learning, the most crucial step is to initialize the number of activation peaks of each feature map in advance to form an initial decision tree. The shallower layers contain more activation peaks, whereas the deeper layers' feature maps have fewer activation peaks, indicating that several child nodes are linked to a parent node.
2. Fusion nodes
  The initial decision tree will be redundant, and the tree needs to be pruned. That is, the activation peaks that lead to the same result are fused into a single activation peak, and the resulting decision tree is the explaining graph of the pre-trained CNN, as illustrated in Figure 14.

Extracting an explanation graph from a pre-trained network is a *post-hoc* interpretability method that does not have any impact on the network structure.

### 3.3. Knowledge map aided zero-shot learning

This section merely considers the convolution and interpretability of the semantic map and briefly introduces the structure and optimization method of graph convolution network (GCN) in combination with node features and link properties. It starts with the description of the general explanation graph and subsequently presents the way of establishing the semantic INN to achieve classify task. Firstly, the explanation graph consists of nodes and adjacent edges, which are classified as directed or undirected graphs based on the properties of the adjacent edges. Secondly, according to the attributes contained in nodes, explanation graphs can be divided into probability graphs and semantic graphs. In the probability graph, each node represents the probability of a presented attribute, and the connected nodes represent a joint probability distribution between two different attributes. In the semantic graph, each node represents a feature vector or a kind of semantic message, and the connected nodes indicate that two features or semantics are available in the whole graph at the same time.

**Figure 15:**
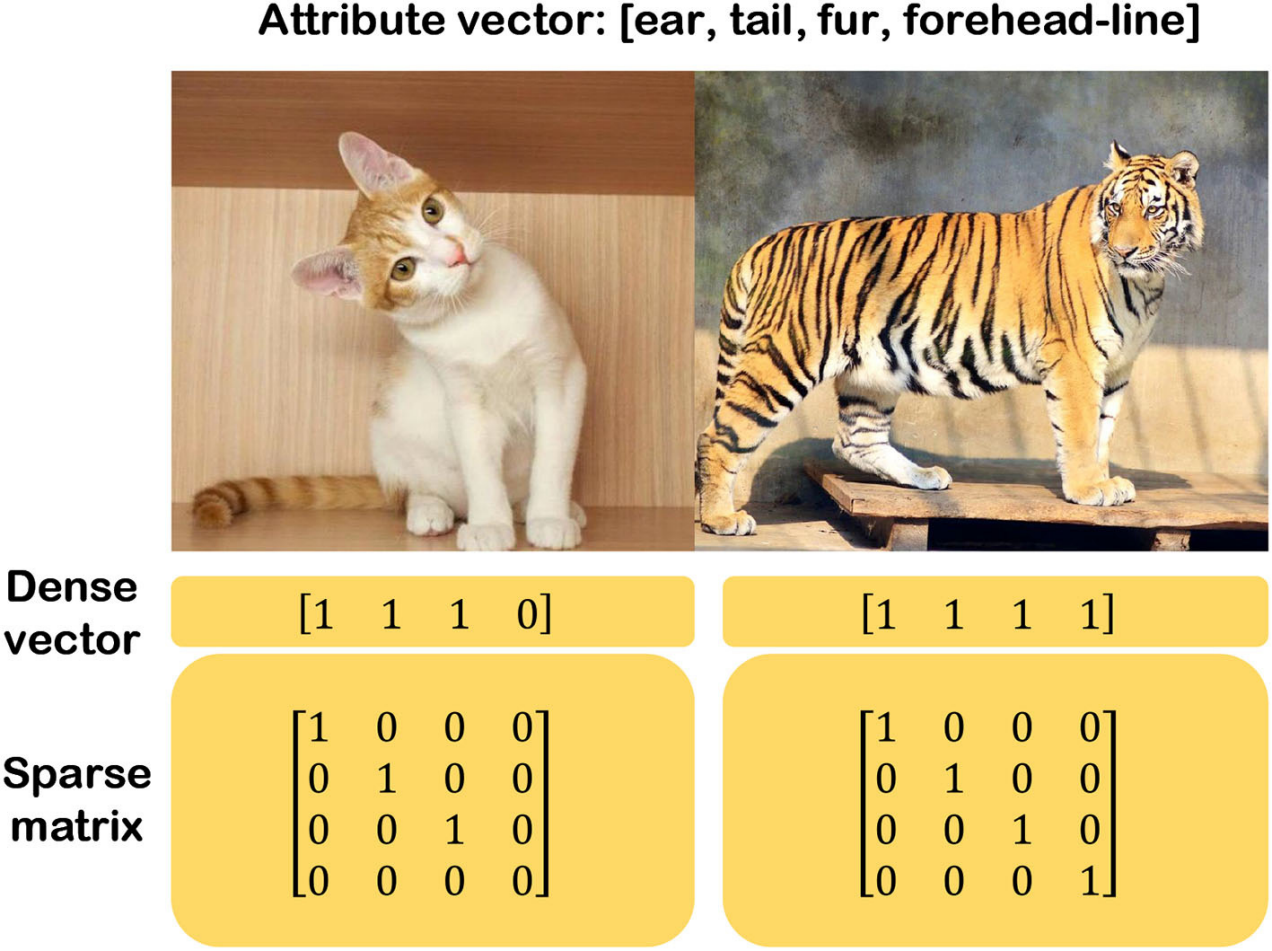
Binary attribute space.

Since the semantic message can be a dense matrix or a sentence, it's crucial to convert this kind of semantic message into a feature vector. For the convenience to separate the grid pixel image from the undirected graph composed of edge nodes, the explanation graph in the non-Euclidean space is collectively named the edge-node graph. Considering an image classification task based on edge-node GCN architecture, the input of GCN is usually a form of word embedding (WE), as engineers always utilize a set of words or phrases to describe the attributes of the feature. The operation of the WE is to convert the words or phrases that are semantic messages of the image into a set of feature vectors and these feature vectors consist of the semantic space. Furthermore, the semantic space contains two categories based on the methods used to construct semantic space: engineering semantic space and learning semantic space. The engineering semantic space is artificially designed by engineers. They describe the target in a unified form based on domain expertise, with multiple meanings for each dimension in the semantic space. However, there are several approaches to constructing the engineering semantic space, and the most common method is the attribute semantic space (Lampert et al., 2009; Palatucci et al., 2009). For example, we can design a simple attribute semantic space for a tiger that consists of ears, tails, fur, and forehead lines. Similarly, the animal “cat” can be described by these attributes without forehead lines. Then, the different attributes of an object can be converted into a vector, where the object prototype has this attribute marked as 1, otherwise marked as 0, as shown in Figure 15. On the contrary, there is also learned semantic space that is obtained by ML methods, that is, it's unnecessary for engineers to manually designate features. However, the semantic vector obtained by ML is no longer interpretable and becomes abstract and incomprehensible to humans.

Following the WE, the next step is to perform convolution on the edge-node graph. Since the manifold space of the edge-node graph distribution does not belong to the Euclidean space, the convolution operation in GCN is very different from that in the pixel graph. The GCN is therefore turned into an operation between the feature vectors in semantic space and the adjacency matrix of the edge-node graph, as illustrated in Equation (35) (Kipf and Welling, 2016).


(35) $$\begin{array}{l} H^{l+1}=\sigma \left({\tilde{D}}^{-\frac{1}{2}}\tilde{A}{\tilde{D}}^{-\frac{1}{2}}H^{l}W^{l}\right), \end{array}$$

where σ(·) is the activation function, *H*^*l*^ and *H*^*l*+1^ represent the feature vectors of the *l*-th level and the *l*+1 level, respectively. The formula above indicates the message passing of nodes between every two layers of an edge-node graph in multi-layers GCN. Besides, the trainable weight matrix *W* is used to control the intensity of nodes in each of the two layers, and $\tilde{D}$ is the degree matrix of the special adjacency matrix $\tilde{A}$ which can be expressed as


(36) $$\begin{array}{l} \tilde{A}=A+I. \end{array}$$

**Figure 16:**
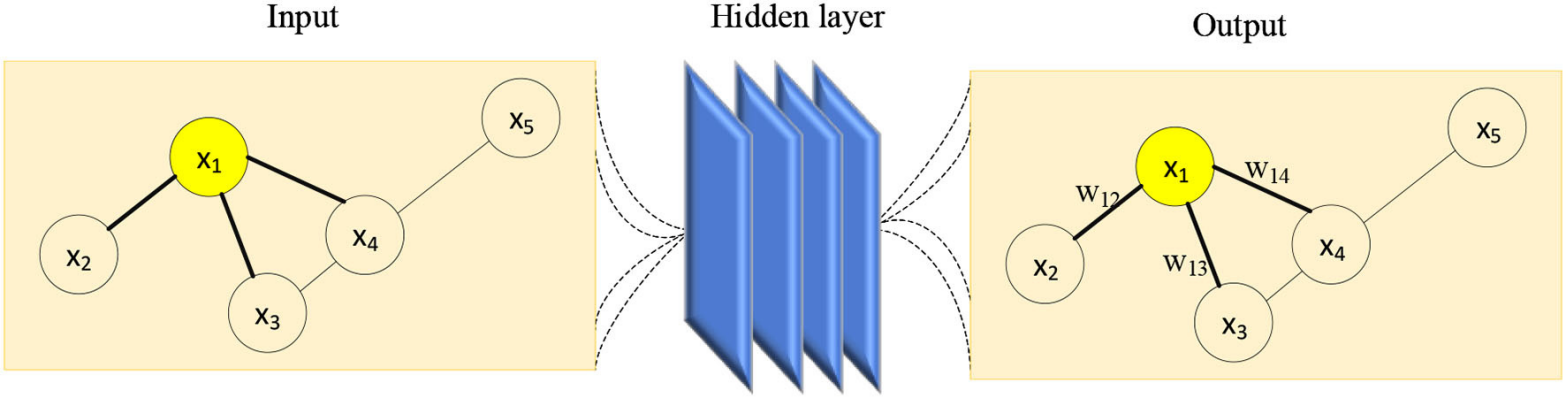
The process of message passing in GCN.

The value in the adjacency matrix *A* indicates whether there is a link between any two nodes in the edge-node graph. Considering the case of the first-order neighbors of one node, the linked nodes are labeled as 1, while the disconnected nodes are marked as 0. Since there must be two nodes connected to the same edge, the adjacency matrix is also a symmetric matrix. The degree matrix *D* is a diagonal matrix, and the values on the diagonal represent the degree of each node, which is determined by the number of edges linking the node. Obviously, the GCN is the process of message passing, and the upper layer feature vector *H*^*l*^ exchanges the message with the deeper layer feature vector *H*^*l*+1^ via the adjacency matrix *A*, where the message is transmitted between specific nodes. To incorporate the node's effect on message transmission, the adjacency matrix is transformed into the form specified by Equation (36). Simultaneously, the trainable weight matrix *W* is employed to manage the process of message transit between two nodes. Following GCN's message traveling through all nodes, the resulting feature vector is denoted as $\tilde{A}H^{l}W^{l}$. Unfortunately, the GCN faces several limitations when it comes to multi-layer transformation. As the number of convolutional layers grows, the output value increases rapidly as well. Thus, in GCN, in addition to the convolution operation, an activation function consistent with DNN is required, and the next level feature vector is denoted by (35). After training the GCN, the linear transformation matrix between layers tends toward a stable value, as shown in Figure 16.

Figure 16 shows the message transmission process of node “1.” Its input graph contains 5 nodes and 5 adjacent edges. Nodes “1” and “4” have three edges each, node “3” has two edges, and nodes “2” and “5” have only one edge each. After the GCN operates on the input graph, the resulting graph retains its structure as the input graph, but the feature vectors and the weight matrix corresponding to the information transmission on the edges have been altered. Considering the input graph given in Figure 16, the adjacency matrices *A* and $\tilde{A}$ of the given edge-node graph, as well as the degree matrix $\tilde{D}$, are represented in Table 2. Using the matrix parameters in Table 2, the resulting feature vector after one time of message transmission can be calculated.

**Table 2:** Adjacency matrix and degree matrix examples.

***A***	** $\tilde{A}$ **	** $\tilde{D}$ **
$\left[\begin{array}{ccccc} 0 & 1 & 1 & 1 & 0\\ 1 & 0 & 0 & 0 & 0\\ 1 & 0 & 0 & 1 & 0\\ 1 & 0 & 1 & 0 & 1\\ 0 & 0 & 0 & 1 & 0 \end{array}\right]$	$\left[\begin{array}{ccccc} 1 & 1 & 1 & 1 & 0\\ 1 & 1 & 0 & 0 & 0\\ 1 & 0 & 1 & 1 & 0\\ 1 & 0 & 1 & 1 & 1\\ 0 & 0 & 0 & 1 & 1 \end{array}\right]$	$\left[\begin{array}{ccccc} 4 & 0 & 0 & 0 & 0\\ 0 & 2 & 0 & 0 & 0\\ 0 & 0 & 3 & 0 & 0\\ 0 & 0 & 0 & 4 & 0\\ 0 & 0 & 0 & 0 & 2 \end{array}\right]$

**Figure 17:**
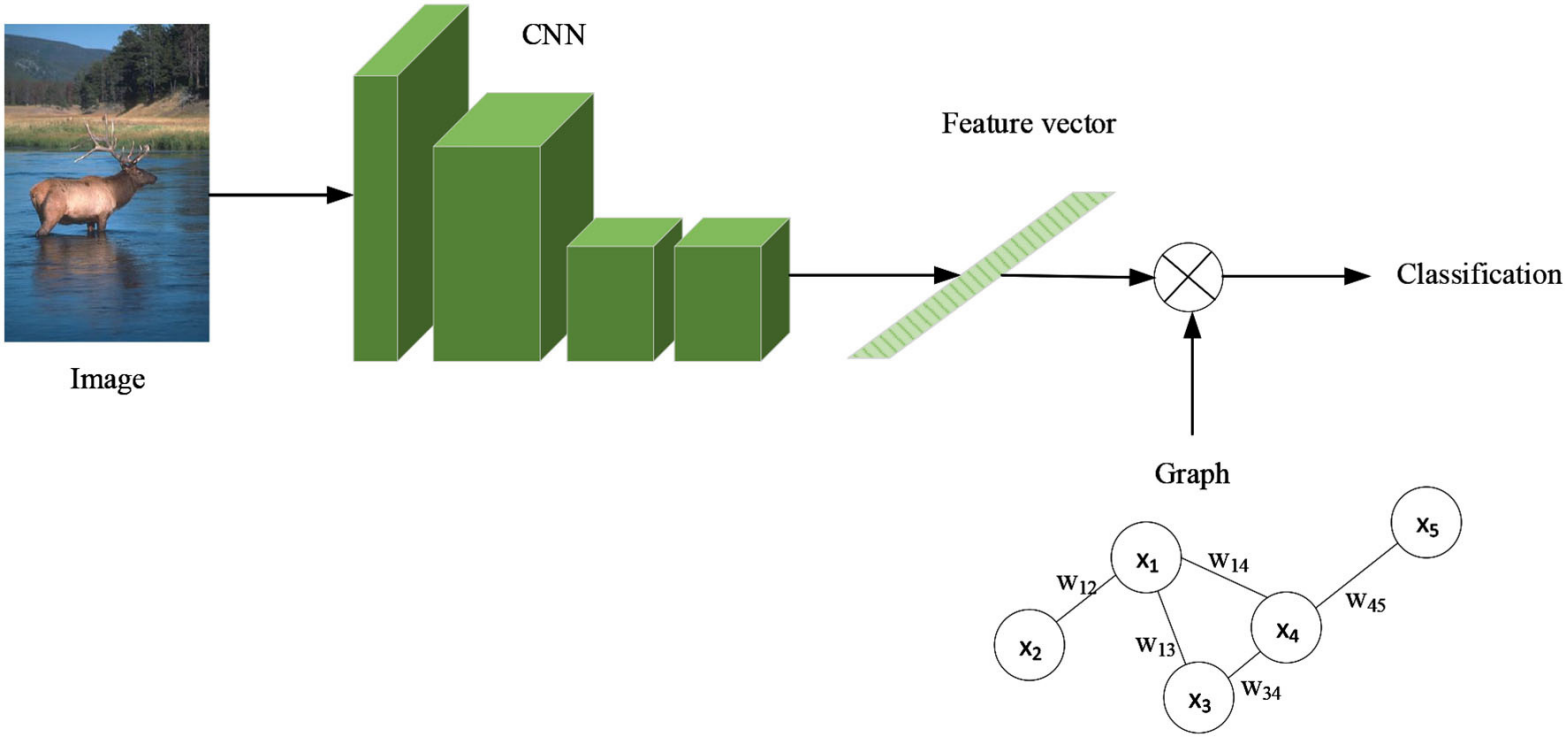
CNN+GCN zero-shot learning solves target classification.

GCN is commonly utilized in social networks, molecular investigation, and natural language processing (NLP). At the moment, the zero-shot learning (ZSL) target classification technique for pixel images that combines CNN and GCN is still under development. In this, ZSL is to solve the classification problem of image objects without the training data of available classes and only provide the description of classes. In addition, it requires computers to be capable to distinguish new objects by learning the way of humans reason without ever seeing their categories. Its fundamental concept is to utilize a pre-trained CNN to extract features from pixel images, then remove the final classification layer and replace it with a GCN to accomplish target classification, as illustrated in Figure 17.

Before training a GCN, the task-based edge node graph structure to be learned must be manually designed. The GCN is then trained in a supervised way to generate the classifier weight matrix, which will be used to replace the classifier in this classification task. In order to add the interpretability of semantic space, Wang et al. (2018b) add the KG to the GCN and combine the semantic attribute space of the edge node graph with the inference described in the KG to accomplish ZSL for unknown category targets.

## 4. Electromagnetic neural networks

There has been considerable research on INN in the field of electromagnetic physics, with the goal of balancing the benefits of classical electromagnetic computing algorithms with DL approaches. However, none of their suggested solutions satisfies a real physical problem. Our objective is to apply the previously mentioned physical model-decomposition INN to solve real-world physical issues. This section introduces and defines the electromagnetic neural network (EMNN), outlines our technique for accomplishing actual electromagnetic physics issues, and describes how the EMNN handles forward and inverse electromagnetic problems.

### 4.1. Demand and challenge of EMNN

**Figure 18:**
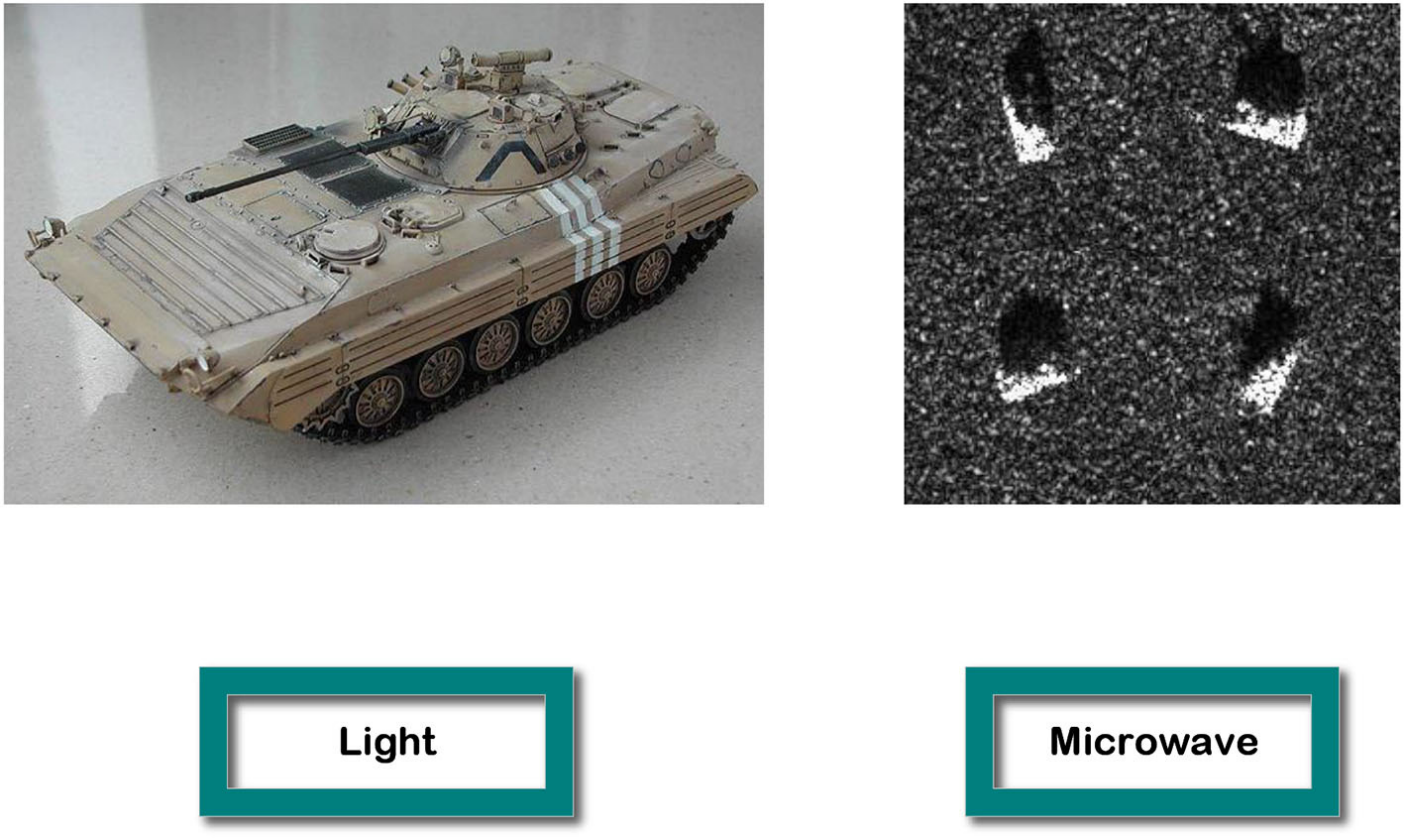
The first picture is the optical image of a tank, and the second is four SAR images of a tank in different orientations (Keydel et al., 1996).

In recent decades, researchers have accomplished the forward and inverse electromagnetic tasks by constructing electromagnetic theoretical models. And, their common requirements and challenges are high computational complexity and slow speed. To overcome these obstacles, DL methods have been gradually used, and they are first used in optical images and then transferred to microwave images. However, the image processing algorithms in the electromagnetic field are very different from those in the optical field because of their different frequency properties, as shown in Figure 18. Additionally, it is challenging to obtain high-quality microwave pictures of objects, and the time cost of data acquisition will be greater than for optical images. Therefore, we propose EMNN to address these issues by embedding electromagnetic scattering models within NNs. Finally, our aim is to achieve fast computation, low complexity, high generalization, and interpretability in EMNN. Furthermore, the NNs can be used to accelerate electromagnetic calculations, and the electromagnetic scattering model is used to enhance the generalization of NNs. As a result, EMNN exhibits the result of rigorous logical reasoning and interpretability.

### 4.2. Definition of EMNN

In comparison to the processing methods of optical images, the electromagnetic neuron theoretical model based on microwave images is developed, which incorporates four critical electromagnetic properties of time, frequency, phase, and polarization. Time is utilized to indicate the time delay *T* of the echo, and different time delays are used to represent the different relative positions of the neuron in space. The relative positions in the multi-dimensional neuron theoretical model can help to relieve the signal oscillation. Frequency-phase-polarization is the element of the signal emission model that describe the electromagnetic properties of the emitted wave, including the signal frequency *f*, the initial phase *P*, and the polarization direction *p*. The specific form is as follows:


(37) $$\begin{array}{l} E_{neurons}=\left[T,P,f,p\right]. \end{array}$$

Multidimensional neurons regarded as an observation matrix can depict a complex electromagnetic environment, which implies that the transmitter at distinct positions can release polarized electromagnetic waves with a special frequency and phase in space. Similarly, the targets in the scene with varying positions, sizes, and materials in the space will stimulate varying responses in this electromagnetic environment. That is, the echo received by the radar contains the electromagnetic scattering of all the targets in the scene, which can be considered as the measurement matrix of the radar.

Furthermore, the growth of neuron models with electromagnetic characteristics involves the implementation of a novel neural information flow transmission method. In comparison to the propagation of electromagnetic waves in free space, the wave function is the fundamental element in the NN's forward propagation process, so Green's function in free space is regarded as the basic solution of the EMNN. By combining it with the expression form of Green's function, the expression of EMNN forward propagation and the gradient descent technique of backward updating can be redefined. Especially compared to standard DL approaches, EMNN can obtain desirable electromagnetic fields or radiation patterns at a faster speed with fewer data.

In this case, the operations of the network layers should correspond to the calculations of the electromagnetic models. For a stricter EMNN, the training parameters in the network correspond to physical properties in the electromagnetic theoretical models. Then, the next step is to decompose the electromagnetic computational algorithm of this problem into iterative steps, which are converted into layers of NNs. Basically, the general electromagnetic model formula is presented as $f\left(s,{\hat{x}}_{i},{\hat{x}}_{j}\right)$ which is exactly known, but some parameters of this formula are unknown. Then, the iterative steps $F_{k}\left(s,{\hat{x}}_{i},{\hat{x}}_{j}\right)$ by solving this EM problem need to be estimated because of the parameters' indeterminacy. These estimated steps can be carried out iteratively by *M* times of addition, or iteratively by *M* times of multiplication, and the specific expressions are shown as:


(38) $$\begin{array}{l} f\left(s,{\hat{x}}_{i},{\hat{x}}_{j}\right)=\overset{M}{\underset{k=1}{\sum }}F_{k}\left(s,{\hat{x}}_{i},{\hat{x}}_{j}\right), \end{array}$$

(39) $$\begin{array}{l} f\left(s,{\hat{x}}_{i},{\hat{x}}_{j}\right)=\overset{M}{\underset{k=1}{\prod }}F_{k}\left(s,{\hat{x}}_{i},{\hat{x}}_{j}\right). \end{array}$$

In the EMNN, it assumes that some iterative steps $F_{k_{g}}\left(s,{\hat{x}}_{i},{\hat{x}}_{j}\right)$ are known and others $F_{k_{n}}\left(s,{\hat{x}}_{i},{\hat{x}}_{j}\right)$ are unknown, which are related to their front steps $F_{k_{p}}\left(s,{\hat{x}}_{i},{\hat{x}}_{j}\right)$. To replace the unknown component of the solution, a special NN is manually designed to fulfill the mapping between the two continuous steps or some layers that can estimate the appropriate physical characteristics. They are written as


(40) $$\begin{array}{l} F_{k_{n}}\left(s,{\hat{x}}_{i},{\hat{x}}_{j}\right)=\overset{M_{p}}{\underset{p=1}{\sum }}N_{p}\left(F_{k_{p}}\left(s,{\hat{x}}_{i},{\hat{x}}_{j}\right)\right), \end{array}$$

(41) $$\begin{array}{l} F_{k_{n}}\left(s,{\hat{x}}_{i},{\hat{x}}_{j}\right)=\overset{M_{p}}{\underset{p=1}{\prod }}N_{p}\left(F_{k_{p}}\left(s,{\hat{x}}_{i},{\hat{x}}_{j}\right)\right), \end{array}$$

where *M*_*p*_ is the number of front modules, *N*_*p*_ is a NN whose mathematical meaning is the mapping between the front part $F_{k_{p}}\left(s,{\hat{x}}_{i},{\hat{x}}_{j}\right)$ and the unknown part $F_{k_{n}}\left(s,{\hat{x}}_{i},{\hat{x}}_{j}\right)$. It's obvious that getting precise front parts is vital for estimating unknown parts. Then, the unknown modules and known parts are restored to the EMNN basic expression, and through numerous iterations, the iterative algorithm to solve the EMNN problems is derived as follows:


(42) $$\begin{array}{l} f\left(s,{\hat{x}}_{i},{\hat{x}}_{j}\right)=\overset{M_{g}}{\underset{g=1}{\sum }}F_{k_{g}}\left(s,{\hat{x}}_{i},{\hat{x}}_{j}\right)+\overset{M_{n}}{\underset{n=1}{\sum }}\overset{M_{p}}{\underset{p=1}{\sum }}N_{p}\left(F_{k_{p}}\left(s,{\hat{x}}_{i},{\hat{x}}_{j}\right)\right), \end{array}$$

(43) $$\begin{array}{l} f\left(s,{\hat{x}}_{i},{\hat{x}}_{j}\right)=\overset{M_{g}}{\underset{g=1}{\prod }}F_{k_{g}}\left(s,{\hat{x}}_{i},{\hat{x}}_{j}\right)\overset{M_{n}}{\underset{n=1}{\prod }}\overset{M_{p}}{\underset{p=1}{\prod }}N_{p}\left(F_{k_{p}}\left(s,{\hat{x}}_{i},{\hat{x}}_{j}\right)\right). \end{array}$$

The formulae above provide the final expressions for iterative addition and iterative multiplication algorithms, respectively. If the final decomposed front module *M*_*p*_ and the unknown item *M*_*n*_ have a single item, the EMNN model defined by the iterative addition algorithm can be condensed into the residual form, and the iterative multiplication procedures can be turned into linear equations.

### 4.3. Applications of EMNN

The EMNN model is proposed to handle actual forward and inverse electromagnetic issues, and it is appropriate for processing electromagnetic signals because of its robustness, speed, and interpretability (Li et al., 2022b; Liu and Xu, 2022; Zhang et al., 2022). This section will explain how to set up an EMNN to handle the problem of positive radiation pattern prediction using coding antennas.

#### 4.3.1. Coding antennas array radiation pattern prediction based on EMNN

**Figure 19:**
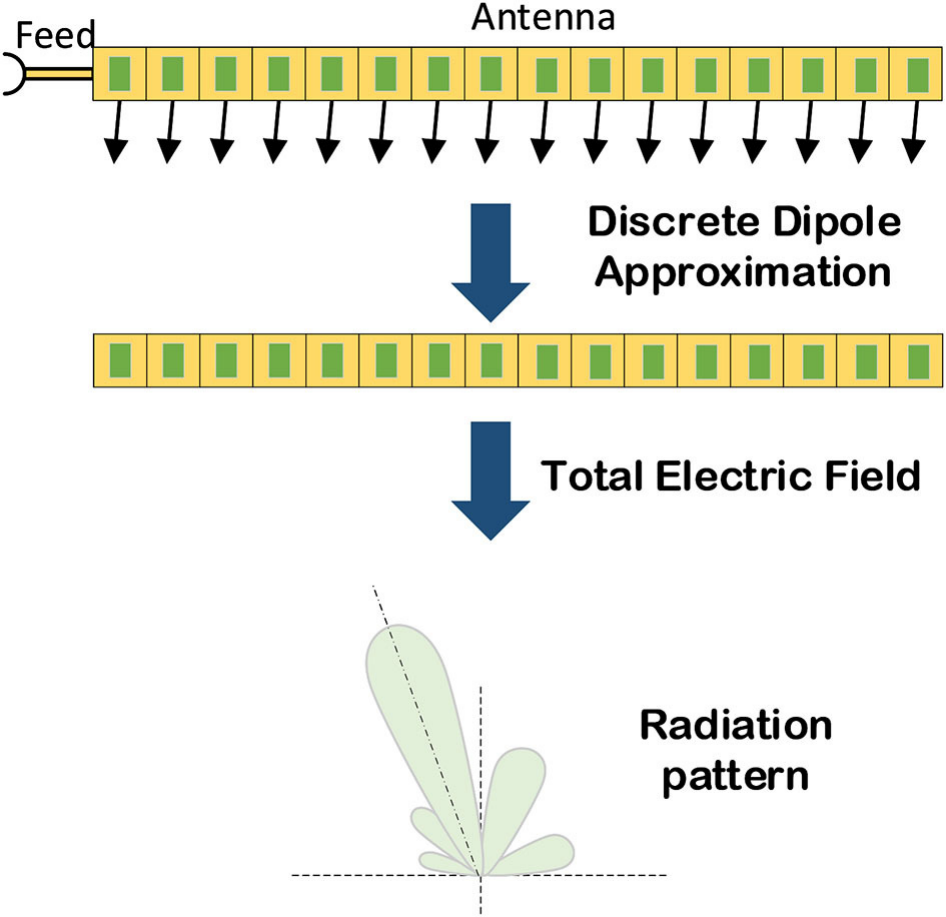
The pipeline of calculating coding antenna array radiation pattern prediction.

The first step of handling the coding antenna array radiation pattern prediction (CARP) problem is to set up the EMNN model, and the process of accomplishing CARP is shown in Figure 19. In addition, the radiation pattern may be described as the multiplicative EMNN model using the discrete dipole approximation (DDA) method. Then it can be consequently reduced to a linear model because there is only one unknown component, which can be deduced as Liu et al. (2021); Li et al. (2022a):


(44) $$\begin{array}{l} E^{tot}=BAH^{inc}. \end{array}$$

where *B* is replaced by the known part *F*_*g*_(θ, *x*_*i*_, *x*_*j*_), and *H*_*inc*_ is replaced by the front part. Then, the transferred matrix *A*, which is the coupling effect between each two antenna elements can be estimated by NNs. Based on the EMNN model, this CARP problem is formulated as


(45) $$\begin{array}{l} f\left(\theta ,x_{i},x_{j}\right)=F_{g}\left(\theta ,x_{i},x_{j}\right)F_{n}\left(\theta ,x_{i},x_{j}\right). \end{array}$$

**Figure 20:**
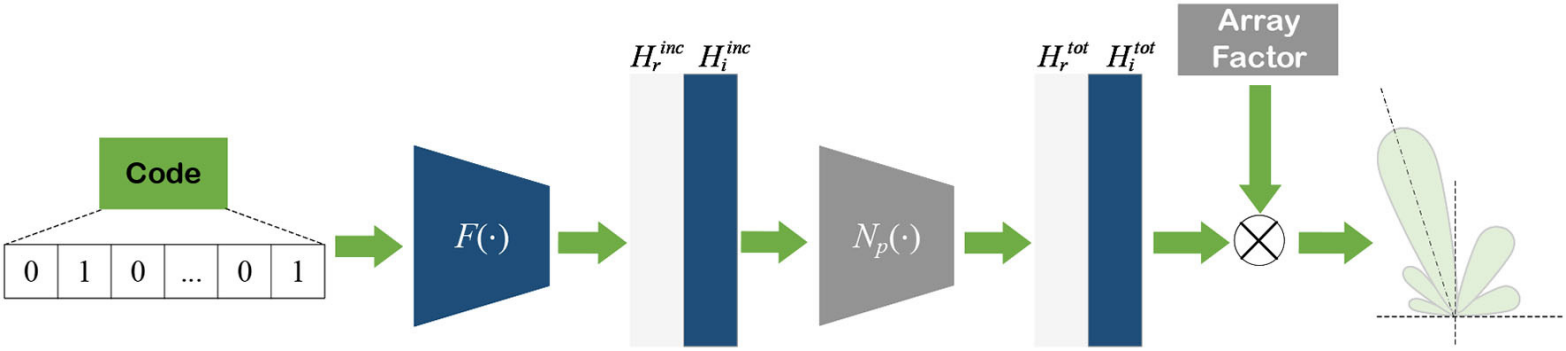
The EMNN structure of solving CARP problem.

In this model, the calculation process of solving the total field is replaced by NN layers, and the expression of the final radiation pattern prediction based on EMNN is shown in Equation (46). This means that it needs to get the incident field as input, and then use some fully connected layers to obtain the total electric field. Finally, the total electric field calculated by the NN is sent to the DDA model to calculate the antenna radiation pattern. The calculation diagram of the EMNN is illustrated in Figure 20.


(46) $$\begin{array}{l} E^{\mathrm{tot}}\left(\theta ,x_{i},x_{j}\right)=BN_{p}\left(H^{\mathrm{inc}}\left(\theta ,x_{i},x_{j}\right)\right). \end{array}$$

## 5. Discussion

In this review, we introduce how to build the EMNN and provide applications for using INNs to solve real-world physical problems. To begin, this paper discusses the limitations of DL methods and model-based techniques in order to demonstrate the significance and necessity of the emergence of INN. Then, the INN is described in two parts, the model decomposition alternative INN and the semantic INN. The former is to explain the traditional models into NNs, which is achieved by transferring reality constraints and formula constraints into layers of NNs. The latter is mainly the “interpretation” of the agent, which builds and analyzes NNs based on semantic features such as vision, logic, and attributes. Finally, considering electromagnetic problems, this paper introduces how to convert the parameters in the electromagnetic model into the NNs' parameters in detail. Below, the strengths, limitations, and prospects of INNs are discussed.

### 5.1. The strengths of INNs

Nowadays, INNs still do not guarantee the reliability of specific tasks, but they facilitate the determination of reliability in the following two perspectives. Firstly, from the perspective of constructing model decomposition alternative INN, combining the traditional mathematical-physical model to build an INN can reduce the network parameters and the network layer design. Secondly, from the perspective of semantic INN, the realization of a posteriori INN after pre-training can help engineers correct errors, which means that engineers can find out from the explanation graph where the network operates in classified tasks or other tasks. To sum up, we compare the INNs with prior DL approaches:

1. Generalizability
  INNs can extract information from the theoretical model of the issue or from the laws governing objective facts and incorporate it into the network's architecture. Then, data independence and the generalization performance of INN are better than those of traditional methods.
2. Trustworthiness
  The black box architecture will not inspire trust, but the semantic INN can display the layers and feature maps of the NNs. INN inductively obtains hidden information from NNs and portrays it as a decision tree. Incorporating visual, logical, and semantic descriptions of the agent's attributes into the decision tree aids in the comprehension of how the network operates. Therefore, the trustworthiness of INNs can be enhanced.
3. Interpretability
  Either alternative INNs based on model decomposition or semantic INNs, both emphasize “interpretability.” The former interprets the theoretical model as a NN, while the latter interprets the NN as a semantic model.

### 5.2. The limitations of INNs

INNs also have some shortcomings, which are closely related to the way they are constructed. Here, the limitations of INNs are divided into the following three points:

1. Model Limitations
  Model decomposition alternative INNs are useful for handling linear problems. When decomposing a theoretical model, if it is linear, it implies that the network generated by the model is also linear. For those issues that cannot be immediately reduced to a linear model, they cannot be transferred into model decomposition alternative INNs.
2. Semantic library limitations
  Implementing an INN by extracting or constructing decision trees can only be applied to relatively common issues and tasks that can disentangle between nodes. And, in order to effectively use the semantic information in the network, it is required to build a massive semantic library, which demands a significant amount of personnel to manually design an expert system.
3. Interpretable Definition
  It's unknown whether the layers or elements can be assigned to physical facts and semantics one-to-one. Furthermore, not all intermediary portions are confirmed using ground truth, making the evaluation of the network's interpretable parameters unfeasible.

### 5.3. The development prospects of INNs

Currently, there is no strict definition of INNs. In this paper, a novel definition of INN is proposed based on a summary of the current research on INNs. A consistent and unambiguous definition may emerge in the future, and the process of creating an INN will be developed progressively. Based on the strengths and limitations of INNs, some future directions are discussed and suggested.

1. Model function expansion
  Constructing a theoretical model that can be represented uniformly. The function of the layer in the NN meets the requirements of the theoretical model calculation while their parameters are unequal.
2. Nonlinear problems expansion
  To decompose the nonlinear issues, this expansion approach starts with standard methods used to address linear problems. The nonlinear model is reduced to these linear formulas that may be substituted by NNs.
3. Semantic extraction expansion
  NNs can automatically extract semantic information from images and generate a semantic library, and the labels associated with these semantic libraries are all visual images that are directly tied to the decision tree. Applying this form of semantic information to INNs will help experts establish a semantic library.

## Author contributions

ZL conceived the study and wrote the manuscript with support from the supervisor FX. All authors contributed to all aspects of the preparation and approved the submitted version.
